# Achievements in Mesoporous Bioactive Glasses for Biomedical Applications

**DOI:** 10.3390/pharmaceutics14122636

**Published:** 2022-11-29

**Authors:** María Vallet-Regí, Montserrat Colilla, Isabel Izquierdo-Barba, Chiara Vitale-Brovarone, Sonia Fiorilli

**Affiliations:** 1 Departamento de Química en Ciencias Farmacéuticas, Facultad de Farmacia, Universidad Complutense de Madrid, Instituto de Investigación Sanitaria Hospital 12 de Octubre i+12, 28040 Madrid, Spain; 2 CIBER de Bioingeniería Biomateriales y Nanomedicina CIBER-BBN, 28040 Madrid, Spain; 3 Department of Applied Science and Technology, Politecnico di Torino, 10129 Torino, Italy

**Keywords:** mesoporous bioactive glasses, local drug delivery, bone tissue regeneration, functionalization, doping with therapeutic ions, osteoporosis treatment, infection treatment, cancer therapy

## Abstract

Nowadays, mesoporous bioactive glasses (MBGs) are envisaged as promising candidates in the field of bioceramics for bone tissue regeneration. This is ascribed to their singular chemical composition, structural and textural properties and easy-to-functionalize surface, giving rise to accelerated bioactive responses and capacity for local drug delivery. Since their discovery at the beginning of the 21st century, pioneering research efforts focused on the design and fabrication of MBGs with optimal compositional, textural and structural properties to elicit superior bioactive behavior. The current trends conceive MBGs as multitherapy systems for the treatment of bone-related pathologies, emphasizing the need of fine-tuning surface functionalization. Herein, we focus on the recent developments in MBGs for biomedical applications. First, the role of MBGs in the design and fabrication of three-dimensional scaffolds that fulfil the highly demanding requirements for bone tissue engineering is outlined. The different approaches for developing multifunctional MBGs are overviewed, including the incorporation of therapeutic ions in the glass composition and the surface functionalization with zwitterionic moieties to prevent bacterial adhesion. The bourgeoning scientific literature on MBGs as local delivery systems of diverse therapeutic cargoes (osteogenic/antiosteoporotic, angiogenic, antibacterial, anti-inflammatory and antitumor agents) is addressed. Finally, the current challenges and future directions for the clinical translation of MBGs are discussed.

## 1. Introduction

Currently, the biomedical scientific community demands new achievements that render a new generation of advanced bioceramics for bone tissue regeneration. Throughout the 21st century, scientific activity has focused on the development of mesoporous bioceramics that combine the unique structural and textural properties of silica-based mesoporous materials (SMMs) with the bioactive behavior of conventional sol–gel bioactive glasses (BGs) [1]. Discovered in the early 1990s, SMMs found initial application in the field of catalysis, based partly on novel principle, due to their hitherto unprecedented inherent features [2,3,4,5,6]. These features comprise: (i) a stable and highly ordered porous network, showing homogeneous pore morphology (hexagonal or cubic) and regular and tunable size (2–50 nm), (ii) a high surface area (~1000 m^2^/g), (iii) a high pore volume (~1 cm^3^/g) and (iv) a silanol-containing surface that can be functionalized with organic components [7,8,9]. It was one decade later when the research group headed by Prof. Vallet-Regí reported for the first time SMMs as drug delivery systems (DDSs) [10]. The results prove the excellent properties of these matrices to load and release in a controlled manner different therapeutic cargoes. This milestone paper marked the beginning of an extensive and exponentially growing research topic on SMMs as DDSs, which currently keeps on producing thousands of publications each year [11,12,13,14,15,16,17,18,19,20,21,22,23,24,25,26].

On the other hand, SMMs showed in vitro bioactive behavior [27], since an apatite-like layer was formed on their surface after soaking in simulated body fluid (SBF) [28]. Nonetheless, despite exhibiting a silanol-rich surface and high surface areas and porosity, SMMs displayed too slow bioactive responses (>30 days) compared with BGs (~3 days). Several strategies were developed to accelerate the bioactive response of SMMs, such as phosphorous-doping, which decreased the bioactive response to 15 days [29,30], or adding small amounts of BGs, which reduced the time of bioactive response to just one day [31]. These pioneering studies introduced SMMs in the field of bone tissue regeneration [32].

**Figure 1:**
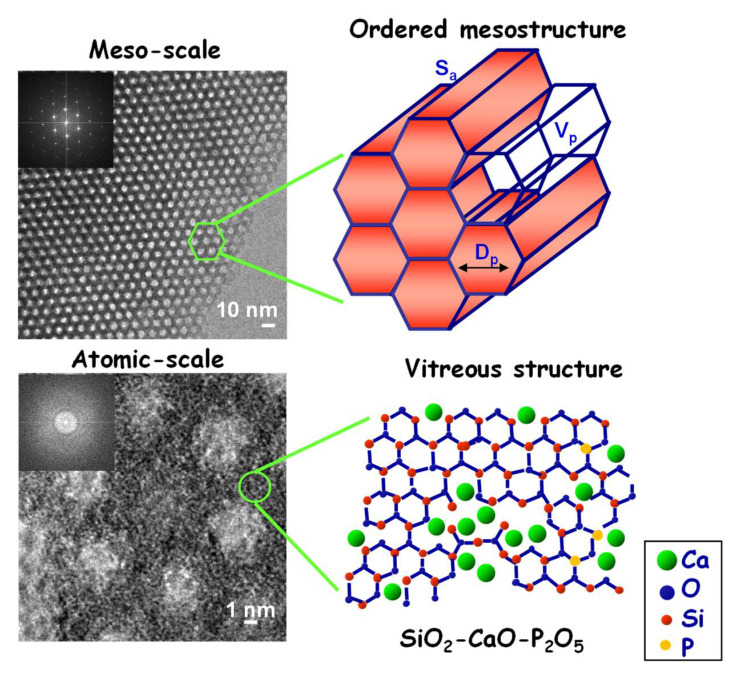
Intrinsic characteristics of MBGs. DP: pore diameter; Sa: surface area; VP: pore volume. Reprinted (adapted) with permission from the reference [39]. Copyright 2010 Elsevier.

However, the major breakthrough in this field was reached through the development of multicomponent ordered mesoporous materials exhibiting the same composition as BGs, known as mesoporous bioactive glasses (MBGs) or “templated glasses” [33]. MBGs constitute a new generation of nanostructured bioceramics displaying unique structural characteristics, being ordered at the meso-scale and disordered at the atomic-scale [34,35]. Amorphous walls, whose composition (SiO_2_–CaO–P_2_O_5_ or SiO_2_–CaO) is similar to that of BGs, separate the channel-like pores that are arranged periodically on highly ordered crystalline structures (Figure 1). Such ordered arrangement of mesopores exhibiting narrow pore size distributions produces textural properties (surface area and pore volume) roughly twice those of sol–gel BGs [36,37,38].

The unique textural and structural properties of MBGs produce superior bioactive responses compared with BGs. Actually, whereas positive bioactive responses of BGs take place in days, in this new family of MBGs, the bioactive responsive happens just in a few hours. Initially, the research efforts focused on determining the pivotal roles that governed the bioactive process in these MBGs. The textural properties of these mesoporous bioglasses predominated over their composition, oppositely to BGs. Additionally, a fine-tuning of the porosity and composition elicited bioactive processes with a higher biomimetic degree [40]. For deeper details on this topic, the reader is encouraged to look up diverse review manuscripts reported in the literature [35,37,39,41,42]. 

The synthesis of these MBGs was not an easy task due to the multicomponent nature of the inorganic system that includes CaO, which hampered the development of robust ordered mesostructures following the conventional methods of SMMs. The research groups headed by Prof. Zhao in 2004 [43] and Prof. Vallet-Regí in 2006 [44] solved this scientific challenge and reported for the first time the synthesis of MBGs in the SiO_2_–CaO–P_2_O_5_ system. This synthesis combined the supramolecular chemistry, using surfactants as structure directing agents, in this case Pluronic^®^ P 123 (EO20-PO70-EO20) (Basf, Europe), with the evaporation-induced self-assembly (EISA) process [45], which was revealed as the cornerstone in the pioneering preparation of MBGs. As in the synthesis of BGs, tetraethyl orthosilicate (Si(OEt)_4_, TEOS), triethyl phosphate (P(OEt)_3_, TEP) and calcium nitrate (Ca(NO_3_)_2_.4H_2_O) were used as SiO_2_, P_2_O_5_ and CaO sources, respectively. The process involved the preparation of a homogeneous solution consisting of the glass precursors and the surfactant in ethanol–water mixture, acidified with nitric acid (HNO_3_) to promote the hydrolysis of TEOS and TEP. Importantly, in the initial solution, the surfactant has a much lower concentration than the critical micelle concentration. Progressive ethanol evaporation increased the concentration of the system, driving the self-assembly of silica–surfactant micelles and their further organization into a liquid crystalline mesophase. After drying and surfactant removal, an ordered mesoporous structure of BGs with high surface area and porosity was obtained.

**Figure 2:**
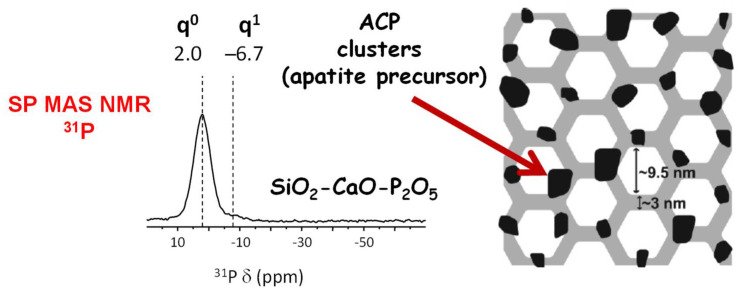
Structural characteristics of MBG. Solid-state nuclear magnetic resonance (NMR) studies have allowed to describe the structural features of these materials, where the simultaneous presence of phosphorous and calcium in the composition forms amorphous calcium phosphate clusters distributed over the entire structure of the glass (red arrow). These clusters have great relevance in the bioactivity process of these glasses, as they act as nucleation centers of the apatite-like layer. Reprinted (adapted) with permission from the reference [51]. Copyright 2008 American Chemical Society.

Additional studies using scanning and transmission electron microscopy (SEM/TEM) coupled to energy-dispersive X-ray spectroscopy (EDS) showed a uniform distribution of Ca, Si and P over the MBG structure [46,47]. This homogeneous distribution, together with the ordered mesoporous arrangement, was used somewhat to justify their improved bioactivity compared with conventional sol–gel BGs, exhibiting heterogeneous distribution of elements [48,49,50]. On the other hand, a systematic study by solid-state nuclear magnetic resonance (NMR) performed by Prof. Edén’s team on MBGs with different compositions proved that the pore wall interior was primarily built by a SiO_2_–CaO glass phase, while P was present as an amorphous calcium orthophosphate, probably distributed as nanometer-sized clusters over the pore wall (Figure 2) [51,52,53,54]. The key role of these calcium phosphate clusters on the structural properties and bioactive response of MBGs has been demonstrated elsewhere [55].

The unique properties of MBGs can be manipulated and controlled at the nanoscale. The nanotechnology applied to bone tissue engineering (BTE) brings up the possibility of fine-tuning the interaction between biomaterial surfaces and cells. Normally, biomaterials exhibiting nanodimensions show higher surface energy and reactivity than micrometric ones [56]. Actually, in the last decade, there has been growing demand of mesoporous bioactive glass nanoparticles (MBG NPs), which can be fabricated by using different synthesis methods such sol–gel [57] or spray pyrolysis [58,59]. Their diminished size allows MBG NPs to be integrated into a wide range of biomedical devices such as cements [60], coatings [61], composite materials [62], scaffolds [62,63] and drug delivery systems [64], making them ideal nanoplatforms for bone tissue regeneration [65,66]. There are multiple approaches to synthesize MBG NPs, which allow modulating not only the inherent properties of MBGs but also their size, morphology and structure, as reported elsewhere [65,66,67,68,69,70]. In this sense, the possibility to obtain the core–shell structure of MBG may be expanded to the biomedical field, such as tissue ingrowth, vascularization, nutrient delivery, etc., as it has been described elsewhere [59,71].

Indeed, MBGs have been receiving remarkable interest as DDSs, mainly owing to their superior and tailorable textural properties—surface area, pore volume and pore size—and their easy-to-functionalize surface. Albeit the term “drug” initially referred to small therapeutic molecules, such as osteogenic, anti-inflammatory, antibiotic or antitumor compounds, over the last two decades, it has been extended to include large biologically active molecules, such as peptides, growth factors, bioactive proteins, enzymes and nucleic acids (DNAs and RNAs) [72]. Moreover, many research efforts have been devoted to preparing a wide range of multicomponent MBGs by doping with therapeutic ions, such as monovalent (Li, Ag, etc.), divalent (Sr, Cu, Co, Zn, Mg, etc.), trivalent (Ce, Ga, Fe, etc.) or tetravalent (Zr) metal ions [36,73,74,75]. The addition of these therapeutic ions provide MBGs not only of well-investigated biological actions, such as osteogenic, antibacterial and osteogenic, but also of new actions such as anti-inflammatory, antitumor, hemostatic, antiangiogenic or cancer preventive, among others [41,76,77]. 

**Figure 3:**
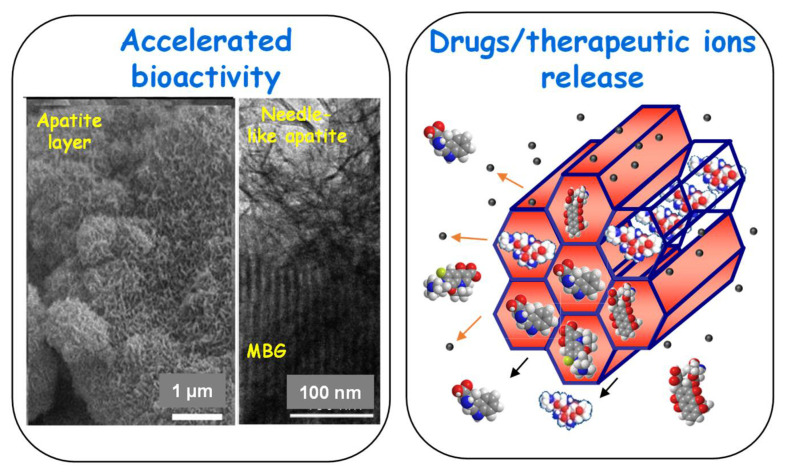
Main properties of mesoporous bioactive glasses (MBGs). Left: improved bioactive behavior. Right: ability to load and release drugs/therapeutic ions in a controlled manner. Black arrows indicate the release of drug molecules; orange arrows show the lixiviation of therapeutic ions.

This key duality of MBGs, accelerated bioactive response and drugs/therapeutic ions release capability, which is schematically depicted in Figure 3, has been the scope of relevant scientific revisions [35,41,66,72,78,79].

It is important to keep in mind the opportunity to functionalize these materials, which permits to adapt their surface to different therapeutic scenarios [35]. Recently, the possibility to design and prepare surfaces able to inhibit bacterial adhesion has irrupted into the field of bioceramics [80]. In this sense, one of the most ground-breaking approaches consist in modifying the surface of MBGs with zwitterionic moieties [81], which is addressed in this review aiming at the design of multifunctional bioceramics. 

Herein, we review the current trends in the design and development of MBGs for biomedical applications. Firstly, the paper describes the role of MBGs in the design and fabrication of three-dimensional (3D) scaffolds for BTE. Another section outlines the different strategies to design multifunctional MBGs, including the incorporation of therapeutic ions in the glass composition and the surface functionalization with zwitterionic moieties to prevent bacterial adhesion. Authors carry out a deep revision of the mushrooming research on MBGs-based local delivery systems of a wide range of therapeutic cargoes. Finally, we tackle the current challenges and future directions for the clinical translation of these bioceramics.

## 2. Mesoporous Bioactive Glasses in the Context of Bone Tissue Regeneration

Bone diseases are one of the main challenges facing today’s society, since bone is the second most transplanted tissue in the world. There are four million operations per year, and this number is expected to increase due to an aging population and a more sedentary lifestyle. BTE has emerged as a promising option to solve this problem [82,83]. BTE uses scaffolds made of biomaterials that mimic bone tissue and serve as a temporary support for cells for their proliferation or differentiation, which is the starting point in regeneration. Moreover, it is also feasible to include growth factors to regulate cellular functions that accelerate such a process. An ideal scaffold for BTE must fulfil the following features[84,85,86]: (i) 3D to mimic bone structure and microenvironment for cells to interact and behave in response to mechanical cues; (ii) biocompatible, i.e., both the scaffold and its degradation products should be non-cytotoxic and do not elicit any undesirable immune response; (iii) biodegradable, i.e., the scaffold should be eventually replaced by the extracellular matrix (ECM) generated; and (iv) hierarchical architecture and osteoconductive activity, i.e., the scaffold must be highly porous and interconnected to facilitate the diffusion of nutrients and oxygen to allow in-growth and outflow of its waste products and cell migration in its interior. Thus, pore sizes in the 600–200 μm range stimulate angiogenesis, whereas pores around 100–60 μm favor osteogenesis; (v) mechanical properties, which should be similar to the bone replaced, since a scaffold that does not mimic the mechano-regulatory forces transferred to the cells under physiological conditions may result in unsuitable cells differentiation and morphology; (vi) cost-effective, simple to produce, scalable and easily sterilized to allow the translation of the scaffold from the theoretical concept to its final clinical form. At present, since bone is a very complex tissue, it is difficult to find a scaffold that collects all the above-mentioned requirements [87,88].

Thus, taking advantage of the intrinsic features of MBGs, numerous research groups are working on the fabrication of 3D hierarchical scaffolds based on these materials [63,89,90]. However, one of the problems in the design of these scaffolds, considering that mechanical support is one of the main functions of the musculoskeletal system, is a proper balance between mechanical properties and porosity, which remains a current challenge [91].

Among the different fabrication techniques to manufacture MBG scaffolds, both the replication using a macroporous template [92,93,94] and the porogen-based methodologies [89] are the pioneering and most widely employed methods. In the first case, polyurethane (PU) foams are generally used as a macroporous template and are impregnated with a MBG sol precursor, giving rise to scaffolds with a hierarchical macro/mesoporous structure after PU template removal by calcination [95]. Concerning the porogen methodology, it uses different templates, such as surfactants (P123 and Pluronic^®^ F 127 (EO100-PO65-EO100) (Basf, Europe)) to produce the mesoporosity and methylcellulose (MC) to yield macroporosity after porogen removal. The obtained scaffolds displayed bone-like trabecular structure with a high porosity (>90 vol%) and interconnectivity [63]. In both methodologies, it is difficult to precisely control the macroporous structure and interconnectivity, and the mechanical strength of the resulting scaffold is severely compromised. 

More recently, 3D printing (3DP) technologies are receiving growing attention by the biomedical scientific community [96]. Such technologies, based on computer-aided design (CAD) and computer-aided manufacturing (CAM), has been used to design and fabricate scaffolds with a precisely controlled macroporous structure and to meet the expanding clinical demand for individualized and customized repair of bone defects with specific shapes [97]. 3DP technology creates the scaffold layer by layer with high reproducibility and with improved mechanical performance, which make its potentially suitable even for load-bearing applications [98,99]. In this sense, 3DP has been effectively used in the fabrication of MBG-based scaffolds for BTE, exhibiting controllable hierarchical porosity and structure [74,100,101]. 

**Figure 4:**
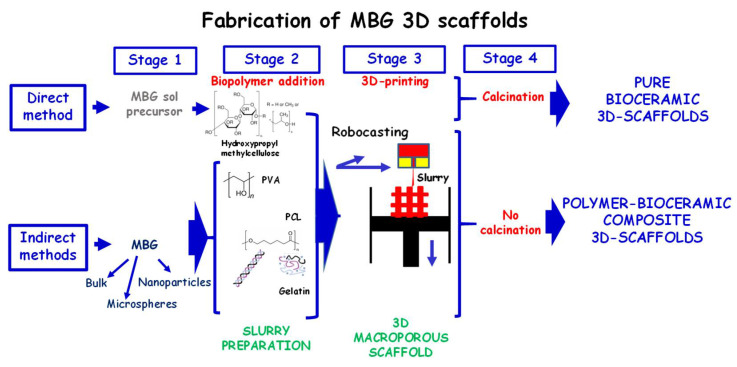
Different approaches for the fabrication of MBG-based 3D scaffolds by rapid prototyping techniques.

Figure 4 displays the main 3DP approaches to fabricate MBG-based scaffolds described in the literature. In all of them, the preparation of a slurry is a key factor, and an exhaustive control of its rheological properties is needed to fine-tune the final structural and mechanical properties of the scaffold [102,103,104,105,106,107,108]. At present, there is not a standardized protocol to prepare a suitable slurry with optimum rheological properties for a continuous and homogeneous extrusion of the 3DP filament. Such factors are generally typical of the slurry (used binder, ceramic particle size, concentration, etc.) and characteristic of the printing process itself (extrusion pressure, temperature, needle tip size, extrusion speed, etc.). In general, there are different approaches based on either direct or indirect methods (Figure 4), whose main difference relies on whether the MBG is previously synthetized (indirect) or not (direct).

**Figure 5:**
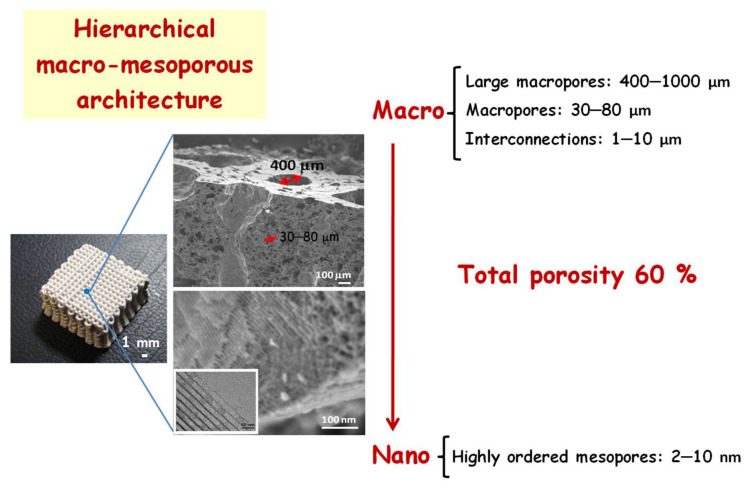
Hierarchical macro-mesoporous architecture of MBG-based 3D scaffolds manufactured by rapid prototyping. Up: SEM micrograph showing the hierarchical macroporosity. Down: high-resolution SEM micrograph and TEM image (as an inset) showing the ordered mesoporous arrangement. Reproduced (adapted) from reference [110]. Copyright 2014 The Royal Society of Chemistry.

In direct methods, the sol–gel technology directly offers the possibility to attain a slurry with suitable extrusion properties by simply acting on aging time of the MBG sol to match appropriate rheological properties for printing. Therefore, an appropriate slurry is prepared by adding a binder as MC to the MBG sol and, subsequently, extruded by 3DP technique to obtain a green scaffold. Thus, to remove the organic material (binder and surfactant) and to obtain the vitreous phase of MBG, the green scaffold is calcined, resulting in pure bioceramic 3D scaffold with a hierarchical structure with a total porosity of 60%. Figure 5 schematically illustrates the different ranges of porosity of 3D scaffolds manufactured by the direct method, where we can observe: (i) giant pores of around 400 μm derived from the porous design itself by the prototyping technique; (ii) macropores between 30–80 μm with interconnections of 1- 10 μm derived from the calcination process of ceramic slurry with MC; and (iii) ordered mesopores of size 2–10 nm derived from the elimination of the surfactant template. The resulting 3D scaffolds offer the advantage of being exclusively composed by MBG glass, allowing an easy control of the degradation process and exhibiting excellent osteoconductive and immunogenic properties [109,110]. Moreover, it was proved that it is possible to enhance the osteogenic effect through an notable increase in osteoconductivity and osteoblastic differentiation by a simple amine functionalization [110].

Recently, inspired by the direct procedure, Wang et al. described the development of MBG-based 3D hierarchical macro-mesoporous scaffolds, exhibiting better mechanical properties than PU-derived MBGs scaffolds. To this aim, they combined sol–gel technology with polymer-photo curing. In this case, the structure-directing agent (F127) was modified to perform both self-assembly and UV curing of the MBG sol precursor during 3DP [111]. The resulting scaffolds displayed enhanced interconnectivity, faster in vitro biodegradation and performance and bone regenerative efficacy in a critical-size rat cranial defect model.

Concerning indirect methods, scientific efforts aimed at combining MBGs with polymer phases, leading to hybrid formulations to engineer multifunctional scaffolds with enhanced physical–chemical, mechanical and biological properties able to promote bone regeneration, also in a compromised clinical context, such as osteoporosis and severe trauma resulting in skeletal non-self-healing defects (Figure 4, indirect method). Such approaches consist in the prior preparation of MBG powder (bulk, microparticles or NPs) and subsequent incorporation into a binder solution (polymer or hydrogel matrix) to form the slurry for extrusion. The concentration of the binder solution as well as the particle size of the bioceramic will determine the properties rheological of the slurry and the final structural/textural properties of 3D composite scaffolds. 

In the last decade, different kinds of materials have been 3D printed to fabricate scaffolds, including natural/synthetic polymers and MBG, with the final aim to mitigate the limitations of the single materials and give full play to their strengths. With this perspective, different research groups have explored hybrid formulations of MBG and poly ε-caprolactone (PCL) to produce printed composite scaffolds for application in the field of BTE [112,113,114,115,116] and drug storage/delivery [106]. In addition, multifunctional 3D scaffolds by printing formulation of MBG dispersed in polyvinyl alcohol (PVA) and polylactic acid (PLA) were reported, which, in addition to having good osteogenic properties, can also act as excellent dosing systems for different drugs [117,118,119,120]. MBG particles, although fully embedded into the different polymer scaffolds, retained their ability to release Ca^2+^ with fast kinetics and, consequently, promote excellent in vitro bioactivity, as well as cell activity, in terms of adhesion, proliferation and differentiation. 

More recently, the 3DP of naturally derived proteins (i.e., silk fibroin and collagen) has been proposed in combination with MBGs to enhance the overall bioactivity and biomimicry of the final composite 3D constructs and, in addition, to better match the resorption kinetics with the deposition of new bone tissue. For this purpose, Du et al. reported the fabrication of scaffolds with high osteogenic ability and favorable mechanical properties by printing layer by layer a formulation of MBG dispersed in silk fibroin (SF) solution [121]. The MBG/SF mass ratio was precisely tailored to obtain a homogeneous paste characterized by a final viscosity matching the printability window. The study additionally reported that the stabilization of the printed composite scaffold was achieved by promoting SF crosslinking in ethanol, favoring the transition from its random coiled form and helix to water-insoluble beta-sheets. Apatite forming ability and degradation rate of the scaffolds were investigated in simulated body fluids, proving that embedded MBGs retained their excellent bioactivity and that the scaffolds showed suitable degradation properties tailored by the crosslinking treatment. In vitro assessment of MBG/SF scaffolds revealed a significantly higher ability to promote human bone mesenchymal stem cell (hBMSC) proliferation and differentiation. Furthermore, the study reported excellent results in vivo, where hBMSCs were loaded into the MBG/SF scaffold and subsequently transplanted into the back of nude mice to test bone regeneration ability. The biological assessment of the transplanted scaffolds revealed efficient expression of bone morphogenetic protein 2 (BMP-2) as well as bone sialoprotein (BSP), showing significantly higher values if compared with reference constructs based on MBG/PCL, further proving better potential in terms of biomimicry.

Composite scaffold with superior biomimetic features can be attained by printing hybrid formulations based on type I collagen, which as the main organic component of the bone extracellular matrix is widely known to have distinctive biological properties, positively influencing cell bio-recognition and adhesion [122]. 

**Figure 6:**
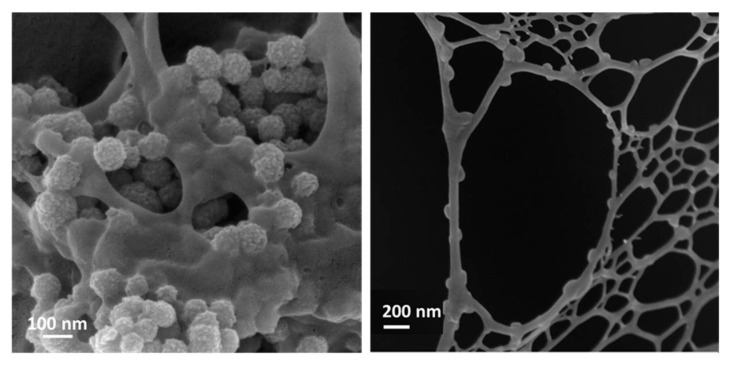
Field emission scanning and electron microscopy (FESEM) images of type I collagen containing Sr-MBG nanoparticles. Reproduced (adapted) from reference [123]. Copyright 2020 MDPI.

Based on these premises, Montalbano et al. described the combination of nanosized Sr-containing MBG and type I collagen to be used as a hybrid biomimetic formulation suitable for the 3D printing of bone-like scaffolds [123]. To mimic the bone composition in the final printed scaffolds, the authors referred to the volume percentages of the organic and inorganic phases in the natural tissue (53 vol % of collagen and 47 vol % of the inorganic phase), which were considered to define the relative amounts of collagen and MB phases for the preparation of the printing formulations. Mesh-like 3D constructs were manufactured while exploiting a supporting gelatin bath and post-printing incubation in genipin solution enabling crosslinking among the collagen fibers and was optimized in order to provide the printed scaffold with suitable mechanical strength and degradation rate, while avoiding the premature release of Sr^2+^ ions. Morphological analyses clearly showed the homogeneous distribution of MBG nanoparticles throughout the collagen-based 3D structure, with an arrangement along the protein fibrils, nicely resembling that of the mineral phase within the native bone matrix (Figure 6). The high bioactivity and the ability to release pro-osteogenic Sr^2+^ ions imparted by MBG, combined with the morphological and physical–chemical stimuli imparted by the 3D printed collagen structures, paved the way for the design of a novel class of multifunctional biomimetic bone-like scaffolds for advanced therapies. 

Currently emerging as an alternative to scaffolding is the use of the injectable materials that can be inserted with minimally invasive surgery for bone regeneration [124,125,126]. 

Fibrin glue (FG), obtained by combining fibrinogen and thrombin solutions, is frequently used as an injectable tissue repairing hydrogel for its excellent biocompatibility, biodegradability and potential osteogenic capacity [127]. However, due to its rapid degradation, low mechanical strength and deficient osteoinductivity, pure FG is generally combined with bioceramic to address these shortcomings [128]. MBG NPs were incorporated into hydrogels to improve both mechanical strength and osteogenic properties, promoting improved cell adhesion and early differentiation of osteogenic cells [129,130]. 

## 3. Design of Multifunctional Mesoporous Bioactive Glasses

Since their discovery in the 2000s, the evolution in the concept of MBGs as multifunctional materials has been a paradigm shift in the field of bioceramics. Thus, the concept of MBGs for clinical applications has evolved from a superior bioactive biomaterial towards a multitherapy system for the management of diverse bone-related pathologies by tailoring their composition and surface functionalization. The possibility to incorporate therapeutic ions into their composition and functionalize their surface via zwitterionization without compromising their inherent bioactivity and drug-delivery capability make MBGs advanced multifunctional platforms for biomedical uses. 

### 3.1. Doping of Mesoporous Bioactive Glasses with Therapeutic Ions

Numerous studies have reported that MBGs can be enriched with various therapeutic metal ions (Sr, Cu, Mg, Zn, Li, Ag, etc.) and loaded with specific therapeutic agents (e.g., osteogenic drugs, antibiotics, chemotherapeutics, growth factors, etc.), achieving controllable loading and release kinetics. Co-delivery of ions and bioactive molecules using a single MBG carrier is highly interesting, as this approach enables synergistic effects and consequently improves therapeutic outcomes compared with the sole drug or ion release. In this review, we discuss the state of the art in the field of mesoporous silica-based materials intended for the co-delivery of incorporated therapeutic ions and drugs with the aim to exert improved osteogenesis, angiogenesis, antibacterial and anticancer properties. The analysis of the literature reveals that ad hoc-designed mesoporous nanocarriers enable the incorporation and release of multiple ions with therapeutically safe and relevant concentrations, achieving the desired biological effects (both in vitro and in vivo) for the targeted clinical applications. 

With specific reference to the field of bone repair, one of the most appealing approaches consists of combining in a single multifunctional bioceramic several abilities, including osteoconductivity (for the guidance of new bone growth) and the capacity to stimulate both osteogenesis (for promoting new bone formation) and angiogenesis (for inducing vascularization). With this challenging perspective, several research groups have explored the development of mesoporous bioceramics enabled by additional biological functions through the inclusion of controlled amounts of therapeutic elements, such as Cu, Zn, Ga, Sr, Li and Ce, while maintaining the necessary bioactivity and biocompatibility, as well as mesoporous structure and high exposed surface area [41].

Very recently, Boccaccini and co-workers authored a review regarding the in vitro/in vivo studies of numerous multifunctional MBGs in the form of NPs, microparticles and scaffolds for the simultaneous release of bioactive ions and therapeutic molecules, highlighting the potential of this dual-delivery approach in terms of complementary therapeutic activities [78]. 

With regard to the stimulation of osteogenesis and concurrent inhibition of osteoclastic activity, the dissolution extract of Sr-containing MBGs ions has been widely proved to provide significant clinical outcomes in terms of bone regeneration potential. In this regard, a study by the authors allowed to clearly elucidate the effect of released Sr^2+^ ions by considering the expression of different types of genes involved in the process of bone regeneration as fundamental markers for the assessment of osteogenic potential [131]. Overall, the in vitro assessment showed that Sr^2+^ release enabled the upregulation of pro-osteogenic genes COLL1A1, SPARC and OPG and the downregulation of receptor activator of nuclear factor kappa-Β ligand (RANKL) of osteoblast-like cells (Saos-2), confirming the therapeutic effect of the element for the stimulation of bone remodeling. With the purpose to widen the overall regeneration potential, Kim and co-workers investigated the co-delivery of Sr^2+^ ion and phenamil (PHE), a small molecule able to significantly enhance osteogenic functions, incorporated into the mesopores of Sr-modified MBGs [132], proving the synergistic effect in terms of enhanced osteogenic differentiation of multipotent stem cells via the bone morphogenetic protein 2 (BMP-2)/small mothers against decapentaplegic (SMAD) protein signaling pathway. In addition to Sr, the incorporation into the MBG framework of other trace elements, such as B and Mg, proved to play an essential role in promoting osteoblast proliferation and in the up-regulation of bone-related genes, also enhanced by the synergistic combination with osteogenic drugs [78]. 

Bone regeneration, especially in the presence of open fractures, also requires effective prevention and fighting the occurrence of bacterial infections that substantially hinder the physiological healing process. In this context, conventional treatments, including systemic antibiotic administration, are often ineffective and may result in quite serious complications leading to extra surgeries. Furthermore, due to the increasing number of antibiotic-resistant bacterial strains, alternative antibacterial agents, such as bioceramics able to release Cu^2+^, Ag^+^ and Zn^2+^ ions, have attracted significant research efforts.

With the aim to combine significant antibacterial properties with excellent bioactivity, Bari et al. studied the incorporation of different amounts of copper into the SiO_2_–CaO framework of MBG NPs [133]. An ultrasound-assisted one-pot sol–gel synthesis allowed to obtain nanoparticles characterized by very high surface area (up to 550 m^2^/g) and containing mesopores throughout their inner structure in the form of a worm-like system. The in vitro analysis, using different bacterial strains, *Escherichia coli* (*E. coli)* (Gram-negative)*, Staphylococcus aureus (S. aureus) and Staphylococcus epidermidis* (*S. epidermidis*) (Gram-positive), demonstrated the significant decrease in the viability of bacteria when cultured with Cu-containing MBG NPs or their ionic dissolution extracts (collected after 24 h of incubation), at variance with the results obtained for MBG without copper. Based on these results, different mechanisms involved in the antibacterial actions of Cu-containing MBG have been envisaged. They are mostly attributed to the release of Cu^2+^ ions for Gram-positive bacteria, at variance with Gram-negative bacteria, whose death resulted not merely in the attribution to the release of metal ions in solution but most likely the small size and the high surface-to-volume ratio of NPs, accounting for the activation of cell death mechanisms. The morphological observation of the biofilm produced by the *S. epidermidis* revealed that Cu-containing MBGs were also able to counteract the biofilm formation and even favor its dispersion, providing a true alternative to traditional antibiotic systemic therapies.

Cu-substituted MBGs also promoted angiogenesis processes, which are known to be key for tissue reconstruction. Paterson et al. successfully demonstrated the combined pro-angiogenic and anti-bacterial effects of Cu-MBG NPs when tested in an advanced 3D infected tissue model [134] and, more recently, El-Fiqi et al. have explored the potential of MBG-based delivery nanosystems for the regeneration of infected tissues through the multiple co-delivery of bioactive Cu^2+^ and antibacterial/angiogenic/osteogenic growth factors [135]. 

As far as antibacterial action is concerned, a post-modification approach to engineer MBG surfaces by incorporating silver has been proposed by Zheng et al. [136], who reported full maintenance of a spherical shape, mesoporous structure, high dispersity, and the apatite-forming ability for Ag-modified particles after surface functionalization. The authors concluded that at variance with the one-step approach to incorporate silver during the synthesis of MBG, resulting in NPs suffering from aggregation and inhomogeneity in size or shape, the post-modification route allowed to retain the overall set of particle features and to incorporate a silver amount of 2.6 mol%. In the study, the synthesized Ag-containing MBG did not cause cytotoxicity towards fibroblasts at the concentration of 1 mg/mL, and this concentration was taken forward for the antibacterial tests. In the study, the antibacterial potential of Ag-modified MBG was successfully assessed for the first time using an advanced 3D-engineered infected tissue model, able to mimic the intricate interactions occurring in vivo, where bacteria invade and interact with other cell types, overcoming the limitations associated to the traditional planktonic bacteria models.

The antibacterial action of released Ag^+^ has been further enhanced by loading antibiotic drugs (i.e., tetracycline) into Ag-modified MBGs, aiming at achieving a synergistic multitargeting function, through the activation of different mechanisms (bacteria membrane rupture and protein inhibition), as described below [78]. 

In addition to imparting antibacterial ability, the composition of MBGs can be tailored to further improve additional therapeutic effects, such as antioxidant properties of particular interest to target oxidative stress related to bone remodeling and disease. With this aim, cerium-containing MBG with a particle size of 100–300 nm has been successfully developed by using an optimized post-impregnation method to incorporate Ce, whose amount was tailored by adjusting the concentration of the precursor solution, while avoiding the formation of by-product cerium oxide NPs (nanoceria) [137]. The impregnation process was optimized by evaluating the influence of temperature and precursor solution concentration on the incorporation yield and nanocluster formation. Once optimized, the impregnation protocol allowed an incorporated amount of cerium equal to 2.8 mol%, and UV–vis and XPS spectroscopy revealed the compresence of both Ce^3+^ and Ce^4+^ species, which accounted for the antioxidant activity. The overall biological characterization evidenced that Ce-containing MBG exhibited anti-inflammatory responses in culture with macrophages and pro-osteogenic activity in culture with osteoblast-like cells, showing great potential as building blocks for a variety of advanced biomedical devices, particularly to target inflammatory bone diseases (e.g., osteoporosis) and delayed bone healing, considering their antioxidant, anti-inflammatory and pro-osteogenic activities.

Ion-substituted MBGs have been also investigated for their anticancer effect induced by the ion release into the tumor microenvironment, also in combination with sustained drug release [78]. Among the elements investigated for this purpose, Ga has attracted interest as a metal ion with anticancer properties, due to its ability to concentrate on highly proliferating tumor cells, which compared with healthy cells have a higher concentration of receptors, becoming an attractive target for gallium ions to bind to [41]. 

Table 1 collects the most representative biologically active ions incorporated into the MBG-based systems (i.e., micro and nanoparticles and scaffolds), with related therapeutic actions as well as in vitro/in vivo biological assessments. 

### 3.2. Zwitterionization of Mesoporous Bioactive Glasses

As an alternative to the excessive use of antibiotics, the design of surfaces that prevent the early stages of an infectious process constitutes a modern and promising alternative in the manufacture of biomaterials [144]. Among the different strategies, the zwitterionization of the surfaces has arisen as a powerful approach in the design of biocompatible bioceramics capable to inhibit bacterial adsorption, which opens new findings in their range of applicability [145]. The efforts focused on designing surfaces that, while inhibiting bacterial adhesion, preserve their bioactive features and allow adequate cell colonization and differentiation, which are key in the regeneration process. In general, the term zwitterionization implies the surface functionalization with both positively and negatively charged moieties, respectively, resulting in surfaces with zero net charge and high hydrophilicity [81]. Originally, surface zwitterionization consisted of functionalization with polymers containing positive and negative charges within the same chain [146,147,148]. Subsequently, it was shown that it is possible to confer zwitterionic properties by functionalization with amino acids such as cysteine and lysine [149,150]. In this case, such surfaces acquire zwitterionic properties depending on the pH of the medium, which is a breakthrough for more specific scenarios depending of the amino acid pKa. However, as the zwitterionic polymers, the amino acid functionalization requires very long processes with many synthesis steps. One of the real breakthroughs in terms of simplicity and cost-effectiveness consists in simultaneous functionalization (bifunctionalization), with organosilanes containing positive and negative charges, respectively. Such strategies allow tailoring the zwitterionic properties by changing the molar ratio between both organosilanes, being able to obtain zwitterionic properties at physiological pH (pH = 7.4) and pH characteristic of a bacterial environment (pH = 5.5) [151]. The bifunctionalization process was first described by Prof. Che and Prof. Terasaki for the synthesis of the amphoteric amino acid bifunctional mesoporous silica for catalytic purposes [152]. This research inspired Prof. Vallet-Regí’s group to design biocompatible SMMs featuring low bacterial adhesion surfaces due to their zwitterionic properties. These surfaces exhibited pairs of -NH3^⊕^/-COO^⊖^ and -NH3^⊕^/SiO^⊖^ groups, covalently grafted through co-condensation bifunctionalization using appropriate precursor silanes [153]. Regarding the possible cytotoxicity of these zwitterionic surfaces, in vitro cell cultures displayed a non-cytotoxic effect and an adequate osteoblastic cell colonization onto their surfaces. The mesoporous arrangements of these materials allowed the loading and subsequent release of different antibiotics, exhibiting a sustained drug release during long periods. In this regard, the functionalization process affected both loading and releasing capabilities due to the different chemical interactions between the drug and the matrix.

The zwitterionization process has also been applied to other bioceramics such as hydroxyapatite in powder, forming 3D-scaffolds [154], and as ceramic coatings onto metallic implants [155]. This has highlighted the versatility of this process for different materials and forms [156]. Recently, mesoporous silica nanoparticles (MSNs) have been also modified by simultaneous direct grafting of hydrolysable short-chain amino (-NH_3_^⊕^) and phosphonate (-PO_3_^⊖^) bearing silane molecules able to provide zwitterionic surfaces under physiological conditions [157].

**Figure 7:**
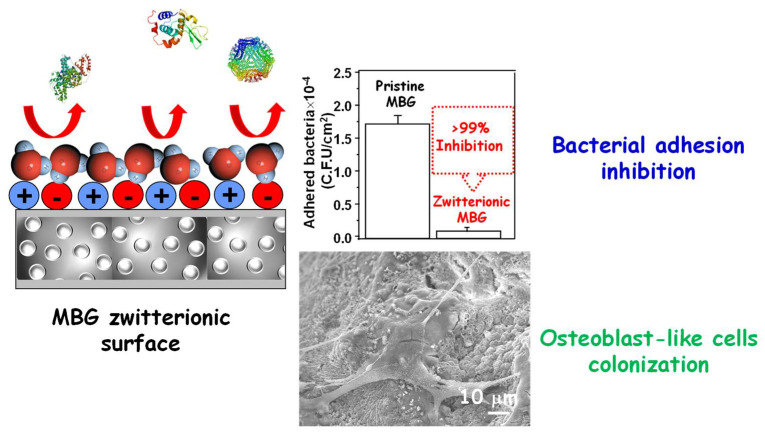
Performance MBG zwitterionic surfaces and biological characteristics, namely bacterial adhesion inhibition and osteoblastic-like colonization. Reproduced (adapted) from reference [145]. Copyright 2018 MDPI.

So far, the described bioceramics, whose surface area has been zwitterionized, display high stability and robustness. However, the challenge has been to create such zwitterionic surfaces onto MBGs, which are highly reactive and where the bifunctionalization process can be compromised. For these purposes, MBG surfaces were previously modified directly post-synthesis with amine groups using (3-aminopropyl)triethoxysilane (APTES) and subsequently functionalized with Lysine (Lys) through glutaraldehyde (GA) linkage through amine groups [158]. The surface anchoring of Lys to the MBG surface provides a zwitterionic nature due to the presence of adjacent amine and carboxylic groups present on the amino acid. After the zwitterionization process, the structural, textural and reactivity/bioactivity features of MBG are preserved. The resulting surfaces notably reduce bacterial adhesion up to 99% compared with pristine MBGs, displaying excellent biocompatibility in pre-osteoblast cell culture. Figure 7 shows a schematic illustration showing the characteristics of zwitterionic MBGs bearing in the surface moieties with positive and negative charge. A histogram showing the bacterial adhesion assay measured through the number of colonies forming a unit attached to such a surface. Finally, a SEM image showing a pre-osteoblast attached to the surface of zwitterionic MBG with its typical fusiform shape emitting anchor-shaped filopodia. 

Additionally, surface zwitterionization of Sr-containing MBG NPs was successfully reported [159] by co-grafting of short-chain organosilanes (aminopropylsilanetriol and carboxyethylsilanetriol), providing a surface bearing the same amount of positively (NH_3_^⊕^) and negatively charged (COO^⊖^) groups. The functionalization route was optimized based on the different reaction kinetics of the two precursors, both in terms of concentration and addition time, and allowed to exhibit the required NH_3_^⊕^/COO^⊖^ zwitterionic pairs and net surface charges close to zero at the physiological pH of 7.4, as revealed by ζ-potential measurements versus pH. In vitro bioactivity was fully preserved by MBG after functionalization, thus demonstrating that the surface ion-exchange reactions promoting hydroxyapatite deposition have not been affected by the presence of the silane moieties. Sr^2+^ release analysis revealed that MBGs after functionalization were able to release the total amount of incorporated Sr^2+^ with kinetics similar to those shown by the corresponding unfunctionalized samples. The concentration of released Sr^2+^ (up to 4.4 ppm) has proved to have the potential to stimulate an enhanced osteogenic response, without inducing any cytotoxic effect [160]. In vitro assay for the evaluation of non-specific protein adhesion demonstrated that the successful grafting of the zwitterionic pairs onto MB surfaces reduced the adhesion of BSA and fibrinogen adhesion up to ca. 85% and 70%, respectively, demonstrating the excellent antiadhesive abilities of the multifunctional developed biomaterials.

## 4. Mesoporous Bioactive Glasses for Local Drug Delivery

**Figure 8:**
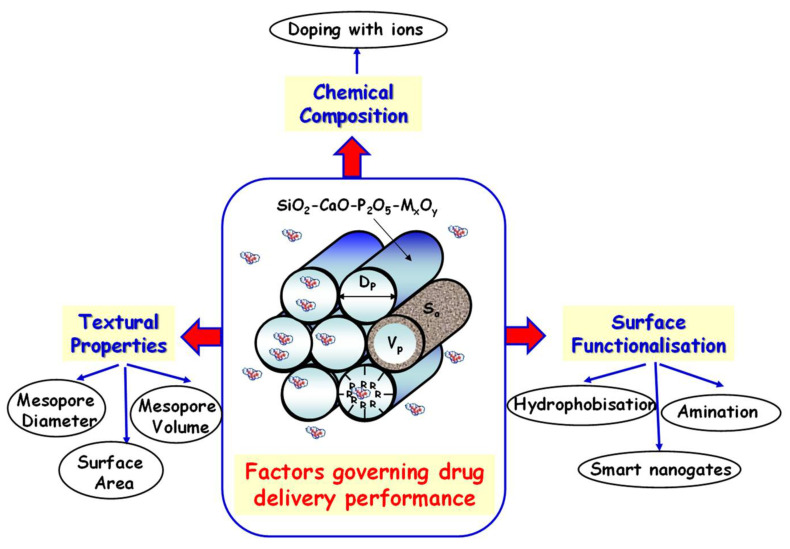
Pivotal factors that govern the performance of MBGs as drug delivery systems. Reproduced (adapted) from reference [21]. Copyright 2021 MDPI.

One of the most important features of MBGs relies on their mesoporous structure, which allows loading different drugs to be released in a controlled fashion, attending to clinical needs. Undoubtedly, this characteristic augurs a bright future in their entry into the clinical practice. As mentioned above, from the structural and textural point of view, and thanks to their easy-to-functionalize surface, these materials resemble SMMs, widely explored as DDSs [11]. Due to this similarity, some of the factors that govern both drug loading and release kinetics from SMMs are extensible to MBGs (Figure 8). However, in the case of MBGs, their highly versatile chemical composition, due to the presence of Ca and P in different proportions, together with the possibility of doping with a wide variety of therapeutic ions, provides a third key factor to take into account in the performance of these materials as DDSs [36,64,78]. 

In this section, we summarize the main research articles focused on MBGs as local drug delivery carriers. It was organized taking into account the main therapeutic action of the loaded (bio)molecules. 

### 4.1. Osteogenic and Antiosteoporotic Agents

As an alternative to the excessive use of antibiotics, the design of surfaces that prevent the early stages of an infectious process constitutes a modern and promising alternative, and MBGs are ideal local delivery platforms for osteogenic and antiosteoporotic agents for synergetic bone tissue regeneration, as summarized in Table 2. Among osteogenic drugs, dexamethaxone (DEX) was chosen by several research groups to carry out loading and release studies using different MBGs. Zhang et al. used DEX as a model drug and investigated its loading and release behavior from different Sr-containing MBG 3D-scaffolds [102]. High drug loading efficiencies (ca. 50% wt.) were ascribed to hydrogen bonding between hydroxyl groups of DEX and the surface of the Sr-MBG scaffolds. Additionally, each type of Sr-MBG scaffold exhibited similar release profiles, with an initial burst effect where 75–85% of the loaded DEX was rapidly released during the first 24 h, followed by a much more sustained release. In another work, Wu et al. loaded DEX into B-containing MBG scaffolds and observed that, although the textural properties (surface area and mesopore volume) of the resulting materials underwent a slight reduction due to B incorporation, the loading and release of DEX were not affected [92]. In vitro assays revealed that delivery of B ions from B-MBG scaffolds for 72 h significantly improved the proliferation and bone-related gene expression (Col I and Runx2) of osteoblasts. Additionally, the further sustained release of DEX over 350 h efficiently enhanced alkaline phosphatase (ALP) activity and gene expression (Col I, Runx2, ALP and BSP) of osteoblasts. DEX and B species release from these MBG-scaffolds supported synergistic in vitro osteogenic proliferation and differentiation. El Fiqi et al. fabricated electrospun fibrous scaffolds of polycaprolactone–gelatin (PCL-GE) incorporating MBG NPs loaded with DEX [161]. In vitro tests in acellular aqueous media revealed that the scaffolds released significant amounts of Ca^2+^ and Si species, whereas the release of DEX was highly sustained. Moreover, the proliferation and osteogenic differentiation of rat periodontal ligament stem cells (PDLSCs) significantly enhanced thanks to the incorporation of MBG NPs into the scaffolds and were synergistically stimulated after DEX loading. The effects on bone regeneration in a rat calvarium model over 6 weeks, investigated by microcomputed tomography and histological stains, demonstrated the improved osteogenic capability of DEX-loaded scaffolds in vivo.

Local drug delivery from MBGs is of foremost relevance in the case of hydrophobic biologically active agents, which in other circumstances experience a broad first-pass effect and exhibit low oral bioavailability. This is the case of some antiosteoporotic agents, such as the ipriflavone drug (IPF), an isoflavone derivative, and estradiol (E2), an estrogenic steroid hormone used via systemic administration in the prevention and treatment of postmenopausal osteoporosis [162,163,164]. In the race towards developing local IPF releasing MBGs-based systems, Vallet-Regí’s team has made significant contributions. In a first approach, they functionalized 85SiO_2_-10CaO-5P_2_O_5_ (mol%) (Si85) MBG with different organic groups, such as aminopropyl (Si85NH_2_), hydroxypropyl (Si85OH), mercaptopropyl (Si85SH) and phenyl (Si85Ph) [165]. The Si85Ph sample exhibited the highest IPF loading (11.7% wt.) and the longest-term release (3% IPF after 10 days of test in a low-polar isopropanol:water medium, in which IPF is highly soluble). These findings accounted for the prevalence of π–π stacking interactions between –Ph groups of the host matrix and the aromatic rings of the guest molecule, over hydrogen bonds between IPF and –NH_2_, –OH and –SH present in the other MBGs. Later on, this research group reported the effect of SiO_2_–CaO MBG hollow NPs loaded with IPF on osteoblast/osteoclast co-cultures [166]. These MBG NPs exhibited a hollow core surrounded by a radial mesoporous arrangement at the shell, which allowed an IPF loading efficiency of 13% wt. Release assays in a low-polar hydroalcoholic medium revealed that only a minor fraction of the total payload was released by diffusion through the mesoporous shell and that most of the drug was retained into the hollow core. The authors pointed out that at the less favorable aqueous physiological environment IPF would be retained for longer periods. Thus, the total IPF release would rely on the MBG NPs degradation which, driven by a great ionic exchange with the surrounding fluids [167,168], would ensure the local long-term pharmacological treatment beyond the initial fast IPF release. In vitro assays in co-cultures of osteoblasts and osteoclasts showed that IPF-loaded MBG NPs induced significant diminution in the osteoclast cell number and resorption activity, without compromising osteoblast proliferation and viability. Very recently, the dual-osteogenic and angiogenic capability of these nanosystems was demonstrated in vitro, which avails their potential use as bioceramics in antiosteoporotic and bone tissue regeneration treatments [169].

On the other hand, Wang et al. developed an innovative delivery technology based on MBGs for the localized administration of the highly hydrophobic E2 estrogen [170]. To this aim, they functionalized MBG NPs with *β*-cyclodextrins (*β*-CDs), whose hydrophobic cavity formed an inclusion complex with hydrophobic E2 molecules, and the resulting NPs were electrospun with SF to produce a nanofibrous mesh with enhanced mechanical stability. A slow and sustained release, where ca. 76% of E2 was released after 3 weeks, reduced osteoclast activity, whereas the presence of MBG NPs in SF stimulated osteoblast proliferation and differentiation.

Nowadays, oral administration of aminobisphonates such as alendronate (ALE) for osteoporosis is characterized by extremely low bioavailability and high toxicity [171]. Wang et al. developed amino-functionalized MBG scaffolds, which exhibited twice-higher loading efficiencies and slower release kinetics than pristine MBG scaffolds [172]. Sustained ALE release promoted osteogenic differentiation of stimulated osteogenic differentiation of bone mesenchymal stem cells derived from ovariectomized rats (rBMSCs-OVX) in vitro and promoted in vivo osteogenesis in an osteoporotic rodent model. 

In other research, Liu et al. investigated radial MBG NPs as local delivery systems of fingolimod (FTY729), an osteoinmmunology drug that promotes osteogenesis [173]. Electrostatic interactions between the negative surface of the NPs and the positively charged FTY729 favored loading and sustained release. Additionally, the Ca^2+^ in MBGs would chelate the drug on the pore wall, hampering fast cargo release. This nanosystem synergistically promoted osteogenesis and inhibited osteoclast in vitro owing to its capacity to promote macrophages polarization towards the anti-inflammatory M2 phenotype. Moreover, its ability to improve bone regeneration was demonstrated in vivo.

Some authors explored the possibility to design MBGs as delivery systems of natural substances for the local treatment of osteoporosis as an alternative to reduce the side effects associated to synthetic drugs [174]. Among natural antiosteoporotic agents, icariin (ICA), derived from the *Epimedium* plants, which exhibits clinical antiosteoporotic activity in postmenopausal women [175], was incorporated into MBGs for local treatment of bone defects [176,177]. Thus, Mosqueira et al. loaded ICA into MBG submicronic microspheres doped with different Sr^2+^ amounts, observing that the higher the Sr content, the higher the ICA loading and the faster the release [176]. ICA and Sr^2+^ release from Sr-MBGs promoted in vitro osteogenic differentiation of bone marrow mesenchymal stem cells (BMMSCs) isolated from osteoporotic adult rats. In another work, Shen et al. developed composite scaffolds of SF and MBG NPs for local ICA delivery [177]. The higher drug-loading rate of 93.3% in the hybrid organic–inorganic scaffold compared with pure SF scaffold (87.2%) accounted for the strong adsorption capacity of MBG NPs. The long-term ICA sustained release from the two-component scaffold (~100% after 24 days) promoted osteogenic differentiation of BMSCs in vitro.

Another osteogenic drug consists on phenamil (PHE), underlined as a powerful small molecule BMP signaling activator, which enhances osteoblasts differentiation and mineralization [178,179]. This drug is fairly stable under physiological conditions, cost-effective, less tumorigenic and has diminished side effects related to overdoses compared with biological therapeutic agents such as growth factors [180]. These reasons motivated Kim and co-workers to load PHE into Sr-doped MBG NPs, as above-commented in Section 4.2 [132]. This nanosystem allowed for synergistic co-delivery of PHE and Sr^2+^ BMP signaling agents, enhancing differentiation and maturation of human MSCs derived from dental pulp in vitro and stimulating osseous-dentinal hard tissue formation in a mal-calcification rat model in vivo. 

Finally, the local delivery of certain osteogenic peptides, proteins and growth factors from MBGs also aroused the interest of the biomedical scientific community. The aim is to increase the in vivo therapeutic efficacy of these biomolecules, which sometimes is threatened due to enzymatic degradation, non-linear pharmacokinetics, poor solubility and stability and poor penetration through biological membranes [181]. For instance, Vallet-Regí and co-workers incorporated the pentapeptide osteostatin (OST), consisting in the fraction 107–111 of the parathyroid hormone-related protein (PTHrP) [182], to confer osteogenic properties to Zn-doped MBGs [112,183]. In a first study, the authors proved the capacity of Zn-MBGs loaded with OST to stimulate osteoblastic cell growth and function [183]. Later on, they developed hierarchical 3D meso-macroporous Zn-MBG scaffolds coated with glutaraldehyde (GA) crosslinked gelatin (GE) and loaded with OST. When decorated with human mesenchymal stem cells (hMSCs), these scaffolds enhanced hMSCs growth and osteogenic differentiation [112]. 

Local delivery of growth factors from MBGs was also addressed by different research groups, aiming at overcoming the limitations of clinically available options [184,185]. Berckman et al. reported a BMP-2 delivery system consisting of MBG microspheres synthesized by an aerosol-assisted spray drying method [186]. Long-term and sustained release of low BMP-2 doses, in vitro cytocompatibility and pro-osteogenic response in human bone marrow mesenchymal stem cells (hMSCs) and bone-forming ability in pre-clinical assays envisioned the potential of this system for translation to clinical routine. Kim et al. reported an alternative approach consisting in enlarging the mesopores of MBG NPs to 6.4–6.9 nm to allow the efficient loading a BMP-2 plasmid DNA (BMP-2-pDNA) into MBG NPs [187]. The BMP2-pDNA/MBG NPs complexes were efficiently internalized (~73%) in rat BMSCs, and most of the cells were transfected to express the BMP-2. Later, osteogenesis of the transfected MSCs was confirmed by the expression of bone-related genes, namely bone sialoprotein, osteopontin and osteocalcin. For in vivo assays, the transfected MSCs were delivered at the local level inside a collagen gel to the target calvarium defects in rats, showing improved bone regeneration. Recombinant human bone morphogenetic protein-2 (rhBMP-2) was also incorporated into MBGs for sustained delivery purposes. For instance, Dai et al. incorporated this growth factor into Ca/Mg-doped mesoporous silica scaffolds (CMMS) [188]. The combined ions and growth factor delivery capability made these scaffolds capable of inducing early osteogenic differentiation in vitro (in rat BMSCs) and ectopic ossification in vivo as early as 2 weeks (in a mouse hind limb muscle pocket model). In another work, Xiao et al. developed MBG nanotubular (MBG-NT) scaffolds, which mimic the extracellular matrix (ECM) structure, as rhBMP-2 delivery systems with optimal dose and minimal side effects [189]. Long-term release of rhBMP-2 from MBG-NT promoted cell proliferation and differentiation, as demonstrated by ALP activity and osteogenic-related gene expression of hBMSCs. Recently, Xin et al. prepared an innovative rhBMP-2 delivery from a composite hydrogel as artificial periosteum [190]. In such a work, rhBMP-2 was covalently linked on the surface of MBG NPs and then photo-crosslinked together with methacrylate gelatin (GelMA). Finally, the resulting GelMA/MBGNs-rhBMP-2 hydrogel membrane was loaded with rhBMP-2 via impregnation. In vitro tests revealed that rhBMP-2 kept releasing for 4 weeks, promoting early osteogenic differentiation. On the other hand, the Ca and Si ions release continued for more than 4 weeks, promoting not only initial cell adhesion but also osteogenic differentiation for longer periods. This hydrogel showed great capacity in long-term osteogenesis and bone tissue regeneration in rat calvarial critical size defect.

**Table 2:** Summary of MBG-based delivery systems of osteogenic and anti-antiosteoporotic agents.

Carrier	MBGs Nominal Composition	Drug	Loading Capacity	Release Behavior	Biological Assays	Ref.
MBG scaffolds	(80 − 2x)SiO_2_-15CaO-2.5P_2_O_5_-xB_2_O_3_ (x = 0, 2.5 and 5 mol%)	Dexamethasone (DEX)	275 µg/g (x = 0) 325 µg/g (x = 2.5) 300 µg/g (x = 5)	Sustained release independent of B-content up to ~100% after 350 h in PBS.	In vitro with human osteoblasts.	[92]
Biopolymer fibrous scaffolds incorporating MBG NPs	75SiO_2_-25CaO (mol%)	DEX	63% wt.	Initial burst release of ~30% within 24 h, followed by an almost linear release after 28 days of test in water.	In vitro with rat PDLSCs. In vivo in a rat calvarium defect model	[161]
Hollow core–shell MBG NPs	79.4SiO_2_-18.1CaO-2.5P_2_O_5_(mol%)	Ipriflavone (IPF)	13% wt.	Initial burst release of 18% within 10 h, followed by a slower release up to 25%. No further release was observed during 7 days in isopropanol: water medium.	In vitro with cocultures of osteoblasts (human Saos-2) and osteoclasts (differentiated RAW-264.7 macrophages)	[166]
Beta- cyclodextrin (*β*-CD)-modified-MBG NPs/silk fibroin (SF) mesh nanofibers	80SiO_2_-15CaO-5P_2_O_5_ (mol%)	Estradiol (E2)	37.99 µg/mg	Sustained release, reaching ca. 76% after 3 weeks in PBS.	In vitro with MC3T3-E1 and pre-osteoclasts RAW 264.7.	[170]
Amino-functionalized MBG scaffolds	80SiO_2_-15CaO-5P_2_O_5_ (mol%)	Alendronate (ALE)	17.1% wt.	Initial burst release of ca. 20% during 24 h followed by sustained release, reaching ~60% release after 280 h in SBF.	In vitro with rBMSCs-OVX. In vivo in an osteoporotic rat model.	[172]
MBG NPs	80SiO_2_-16CaO-4P_2_O_5_ (mol%)	Fingolimod (FTY720)	9.33 µg/ mg *	Initial burst release of ~35% followed by a sustained release, reaching ~95% after 300 h in NaCl 0.9%, pH = 7.4.	In vitro with mBMSCs and RAW 264.7. In vivo in rat femoral condyles defect model.	[173]
MBG submicronic microspheres	80SiO_2_-(16-x)CaO-4P_2_O_5_-xSrO(x = 0, 5 and 10 mol%)	Icariin (ICA)	6.89 %wt. (x = 10) * 5.53 %wt. (x = 5) * 4.21 %wt. (x = 0) *	Initial fast release during 24 h followed by sustained release up to 21 d in SBF. Faster release rate and greater maximum drug release as the Sr-content increases due to the greater mesopore size.	In vitro with BMMSCs isolated from adult rats with and without osteoporosis.	[176]
Silk fibroin (SF)/MBG NPs scaffolds	80SiO_2_-16CaO-4P_2_O_5_ (mol%)	ICA	N.A	Sustained and slow release up to ~100% after 24 days in PBS.	In vitro with BMSCs.	[177]
MBG NPs	85SiO_2_-10CaO-5SrO (mol%)	Phenamil (PHE)	29% wt.	Initial burst release of 22% at 1h, followed by sustained release of 53% after 5 h, ~80% after 30 h and ~90% after 10 days in a Tris-HCl buffered solution, pH = 7.4.	In vitro with human MSCs from dental pulp. In vivo in rat model involving mal-calcification conditions.	[132]
MBGs in bulk compacted into disks	(80-x)SiO_2_-15CaO-5P_2_O_5_-xZnO (x = 0, 4 and 7 mol%)	Osteostatin (OST)	0.8, 0.9 and 0.5 µg/g for x = 0, 4 and 7, respectively	OST release profiles were independent of the Zn-content. Fast release of ~75% after 24 h, reaching total release after 48 h in PBS.	In vitro with mouse pre-osteoblasts MC3T3-E1.	[183]
GA-Gel coated MBGs scaffolds	(80 − x)SiO_2_-15CaO-5P_2_O_5_-xZnO (x = 0, 4 and 5 mol%)	OST	0.52, 0.71 and 0.62 µg/g for x = 0, 4 and 5, respectively	OST release profiles were independent of the Zn-content. Fast release of ~60% after 1 h, ~90% at 24 h and ~100% at 96 h	In vitro with hMSCs.	[112]
MBG microspheres	85SiO_2_-15CaO (mol%)	BMP-2	66.7 µg/mg	Prolonged and sustained low-dose BMP-2 release without an initial burst up to 14 days in either PBS or tris-HCl buffer, pH = 7.4.	In vitro with primary hBMSCs. In vivo in a femoral osteotomy model of compromised healing in female rats.	[186]
MBG NPs	85SiO_2_-15CaO (mol%)	BMP-2-plasmid DNA (BMP-2-pDNA)	3.5 % wt.	Sustained BMP-2-pDNA release up to 2 weeks in PBS.	In vitro with rat BMSCs. In vivo in critical-sized calvarial defects in rats.	[187]
MBG nanotubular scaffold	80SiO_2_-15CaO-5P_2_O_5_ (mol%)	rhBMP-2	24.7 ng/mg (MBG-NT100) and 184.3 ng/mg (MBG-NT100) for rhBMP-2 initial concentration of 100 ng/mL and 500 ng/mL, respectively.	Initial rhBMP-2 burst release of ~63% (MBG-NT100) and ~34% (MBG-NT500) for 3 days, followed by sustained release ≥80% up to 28 days in PBS.	In vitro with hBMSCs.	[189]
GelMA/MBG NPs-rhBMP-2 disk-shaped membrane	80SiO_2_-16CaO-4P_2_O_5_ (mol%)	rhBMP-2	34.5 ng per disk	Long-term sustained release with an initial rhBMP-2 release of 38% after 2 days and ~69% after 28 days in PBS.	In vitro in BMSCs cultures. In vivo in critical bone defect model of the rat skull.	[190]

* Values calculated from the drug loading efficiency (%) and taking into account the loading conditions reported for each research study. Abbreviations: BMMSCs: bone marrow mesenchymal stem cells; GA-Gel: glutaraldehyde (GA) crosslinked gelatin; GelMA/MBG NPs-rhBMP-2: MBG NPs covalently grafted with rhBMP-2 and photo-crosslinked with methacrylate gelatin; methacrylate gelatin; hBMSCs: human bone marrow mesenchymal stem cells; hPDLFs: human periodontal ligament fibroblasts; MC3T3-E1: osteoblastic-like cells from mouse; N.A.: Not available. PDLSCs: periodontal ligament stem cells; RAW 264.7: monocyte/macrophage-like cells derived from BALB/c mice; RBCs: red blood cells; rBMSCs-OVX: bone mesenchymal stem cells derived from ovariectomized rats; Saos-2: human osteosarcoma cell line.

### 4.2. Angiogenic Agents 

Taking into account that bone is a highly vascularized tissue, bone regeneration strategies should focus not only on promoting new bone formation but also on stimulating angiogenesis. The ionic degradation products from MBGs in the biological environment has been proved to stimulate the production of vascular endothelial growth factor (VEGF), provoke VEGF gene expression in vitro and increase the angiogenesis process in vivo [66]. The combined action of local delivery of angiogenic agents such as growth factors from MBGs becomes a powerful and efficient y bone tissue regeneration. Delivery of VEGF from MBGs has been reported [191,192,193]. Very recently, Schumacher et al. described an alternative approached based on the immobilization of VEGF in native state to MBGs using a binding peptide [194]. In vitro assays based on the formation of endothelial cells in response to this material proved that the growth factor activity was preserved after the immobilization process. The appropriate angiogenic stimulation ability of this system avails VEGF immobilization as a promising strategy to circumvent the drawbacks of insufficient neovascularization in the regeneration of large bone defects.

### 4.3. Antibacterial Agents

Bone regeneration remains a clinical challenge that demands the combination of diverse therapeutic approaches. In this sense, the development of osteogenic and antibacterial biomaterials to fight potential infection processes emerging from the surgery itself is receiving growing interest. MBGs are receiving growing interest owing their great versatility, in terms of composition and drug loading capacity, to design advanced antibacterial therapies, which has been the scope of different review articles [195,196]. 

Herein, we summarize the recent advances committed to develop MBGs delivery systems of antibacterial agents, albeit some of the MBGs incorporate therapeutic ions with well-known bactericidal activity (e.g., Zn^2+^, Ag^+^, Cu^2+^, etc.) into their composition. Thus, in vitro assays in acellular physiological solutions (pH = 7.4, 37 °C) with MBGs exhibiting different compositions and/or functionalizations were carried out to evaluate loading and release performances of different antibiotics, such as amoxicillin (AMX) [197], levofloxacin (LVX) [198], moxifloxacin (MOX) [199], teicoplanin (TEC) [200], triclosan (TCS) [200] or vancomycin (VAN) [200]. In addition, deeper studies on the biological response of MBG-based antibiotic delivery systems were in vitro and/or in vivo evaluated, as discussed below. 

Lee et al. reported the preparation of Ag-doped MBG NPs (85SiO_2_-10CaO-5AgO wt%) as carriers of tetracyclin (TC) [143]. The high TC loading capacity of 33 %wt. was due to the high mesoporosity of Ag-MBG NPS and to the TC properties, since this positively charged drug shows high affinity to Ca^2+^ in the MBG. The nanocarriers released in a sustained fashion multiple ions (Ag^+^, Ca^2+^ and Si species) and TC drug therapeutically relevant doses. In vitro antibacterial assays in co-culture *E. coli*, *Streptococcus oralis* (*S. oralis), Enterococcus faecalis (E. faecalis)* and *Streptococcus mutans* (*S. mutans*) planktonic bacteria indicated that bacteria membrane binding of NPs allowed efficient delivery of Ag^+^ and TC to the intra-bacterial space, enhancing antibacterial effects of Ag^+^ (membrane rupture) and TC (protein synthesis inhibition). This complementary antibacterial function inhibited bacterial growth of all tested strains (even the relatively Ag^+^-resistant *S. mutans* and *E. feacalis*) at a concentration of 213 µg/mL. In vitro assays proved that NPs internalized into human dental pulp stem cells (hDPSCs) (~90%) with excellent viability, eventually promoting odontogenic differentiation due to Ca^2+^ and Si-species released.

In vivo assays in an infected dentin-pulp defect model in rats demonstrated that the administration of the therapeutic nanosystem produced total bacterial eradication and efficiently regenerated tissues after 6 weeks. In a very recent work, Jiménez-Holguín et al. developed hollow bioactive glass NPs (HBNPs) of composition 79.5SiO_2_-(18 − x)CaO-2.5P_2_O_5_-xCuO (x = 0, 2.5 or 5 mol%) as loading and release systems of the antibiotic danofloxacin (DFX) [68]. The DFX loading capacities were 4.2, 5.4 or 5.0 for x = 0, 2.5 or 5, respectively. The adsorption of DFX is similar in the three compositions, being slightly higher in those doped with Cu. The incorporation of Cu^2+^ into the MBG promoted DFX loading and sustained release rate, which was explained by means of cation–drug interactions, as described in the literature for Cu^2+^ and certain drugs [79,201]. The DFX-loaded HBNPs produced no cytotoxic response in MC3T3-E1 pre-osteoblasts cultures, even after 3 days of incubation. Finally, this nanosystem showed a synergistic bactericidal effect against *E. coli* and *S. aureus* bacteria in sessile, planktonic and biofilm due to the combined action of Cu^2+^ and DFX. In another work, the same research group described the development of Zn-containing MBG scaffolds coated with GEL as carriers of different independent antibiotics, namely, rifampicin (RIF), VAN, levofloxacin (LVX) and GEN [142]. Molecular modeling, considering antibiotic size and host–guest interactions, explained the different experimental loading efficiencies. Synergistic bactericidal action due to the release of Zn^2+^ together with the antibiotic (LVX or VAN against *S. aureus* and LVX or GEN against *E. coli*) was evidenced throughout the eradication of bacteria in both planktonic and biofilm state. High efficiency using minimal drug doses would contribute to reduce the risk of antibiotic resistance mechanisms.

### 4.4. Anti-Inflammatory Drugs

Bone-related diseases are frequently associated with inflammatory processes, and therefore the possibility of locally delivery of anti-inflammatory drugs into MBGs would be an added value [202]. In this sense, different MBG compositions together with diverse anti-inflammatory agents have been formulated. It is important to remark that even the ionic dissolution products of MBGs exhibit in vitro anti-inflammatory properties, as it was reported for conventional sol–gel bioglasses [203]. 

Chitra et al. incorporated acetaminophen (ACE) and ibuprofen (IBU) into Cu-containing MBGs and evaluated release profiles, showing first-order Korsmeyer–Peppas and Higuchi, which potentially up-regulate the healing properties in dental applications [204]. On the other hand, aspirin was loaded into SF/MGSs composite scaffolds, providing an optimal in vitro drug release over 14 days. Moreover, in vivo assays in mice calvarial defect evidenced the synergistic role of MBGs and aspirin in the bone regeneration process [205]. Very recently, Mo et al. reported the incorporation of naringin (NG), a natural flavonoid with potent antioxidant, anti-inflammatory and antiapoptotic effect, into MBG NPS to facilitate efficient bone regeneration through the regulation of macrophages polarization [206]. To promote NG loading, MBGs were functionalized with β-CD, achieving sustained release within 6 days. In vitro studies revealed that the nanosystem promoted the transformation of macrophages to the M2 phenotype, inhibiting macrophage inflammatory responses. In addition, the induced local immune microenvironment synergistically facilitated osteogenesis and inhibited osteoclastogenesis. In vivo assays in a rat femoral model revealed the synergistic effect on the immunomodulation of osteogenesis and osteoclastogenesis owing to both MBGs and NG. 

### 4.5. Antitumor Drugs

During the last few years, the idea of employing MBGs as delivery system of antitumor drugs has been gaining growing attention. The reason obeys to the possibility of combining bone tissue regeneration capability with anticancer treatment in a unique multifunctional platform [79]. Huge research activity has been committed to evaluate the capability of MBGs as delivery systems of antitumor drugs. It can be classified in two main groups. The first one deals with tuning the host–guest MBGs–drugs interactions to achieve higher control over drug loading and release kinetics and the incorporation of stimuli-responsive elements to allow on-demand cargo release. Among antitumor drugs, the most frequently loaded has been doxorubicin (DOX) [207,208,209,210,211], scarcely imatinib [212], curcumine [213] and daunomycin [214]. 

The second one relies on the new developments aimed at evaluating the interaction of MBGs loaded with different antitumor drugs, such as DOX [215,216,217,218,219,220], camptothecin (CPT) [221], silibinin [222,223] and mitomycin C [224], with diverse cancer cells.

Although the number of publications into this research landscape has experienced extraordinary expansion in the last few years, remaining challenges must be overcome before introducing these bioceramics into clinical trials. 

## 5. Conclusions and Outlook

MBGs featuring similar compositions to conventional bioactive glasses and resembling the structural and textural properties of mesoporous silica materials make them singular bioceramics for local drug delivery and bone tissue regeneration. 

Since their discovery at the beginning of the 21st century, MBGs as multifunctional bioceramics have received growing attention by the biomaterials scientific community. Pioneering research efforts focused on the design and fabrication of MBGs, whose composition, textural and structural properties produced optimal bioactive responses. More recently, MBGs are conceived as multitherapy systems for the treatment bone-related pathologies by the fine-tuning of composition and surface functionalization. 

The possible incorporation of therapeutic ions into the MBGs structure opens up a huge range of opportunities to design versatile and custom-made bioceramics that fulfil the highly demanding clinical requirements of bone tissue engineering. Thus, depending on the specific case, personalized therapies allow not only to address the bone regenerative process but also to treat other complex pathologies such as osteoporosis or cancer.

The feasibility to carry out surface functionalization of MBGs with zwitterionic moieties provides a benefit in the prevention of bone implant infection. Paving the way towards their future clinical translation, a mushrooming expansion of the research focused on the design and fabrication of MBGs-based 3D scaffolds. Among the different technological approaches to manufacture 3D constructs suitable for bone tissue engineering, increasing research efforts have been devoted to 3D printing techniques, which allow the design of customized scaffolds with highly controlled microarchitecture and macropore morphology. Nonetheless, despite the exceptional capacity to prompt bone tissue regeneration, due to their osteoconductive properties and their capability to stimulate osteogenesis and angiogenesis, pure MBGs bioceramic scaffolds exhibit noteworthy drawbacks, which are mainly ascribed to the poor mechanical properties of the constructs. Significant efforts have been committed to fabricate composite scaffolds by combining MBGs and polymer phases that provide adaptive mechanical and shape capacity. In addition, wide research activity has demonstrated the multifunctionality of MBGs which, together with their bone regeneration capacity, allow the local release of drugs (osteogenic, angiogenic, antibacterial, anti-inflammatory or antitumor), whose effect can be powered by the ionic degradation products from MBGs.

Albeit intense scientific activity has been reported by many research groups all over the world, to the best of our knowledge, no system based on MBGs has received approval by the U.S. Food and Drug Administration (FDA). The major obstacles hampering the bench-to-bedside translation are the lack of pre-clinical and clinical studies. Actually, most studies have been carried out in vitro and in vivo, these last ones in small animal models, and deeper studies need to be conducted on large animal models, which are much more human-like.

Finally, the singular properties of MBGs foresee a triumphant entry into the clinical arena, providing efficient treatments to address the complexity of the bone regeneration process. This great challenge requires interdisciplinary approaches, involving collaboration between chemists, material scientists, pharmaceutics, biomedical engineers, biologists/microbiologists and clinicians.

## Figures and Tables

**Table 1:** Overview of selected MBG-based systems containing biologically active ions and their exerted therapeutic actions.

Material	Ion	Therapeutic Action	Studies	Ref.
MBG NPs	Cu^2+^	Antimicrobial Angiogenic	In vitro bacterial viability Biofilm disaggregation/dispersion	[133]
MBG NPs	Cu^2+^	Angiogenic Antibacterial	Aortic Ring/CAM assay Infected 3D tissue model (*S. aureus* and *Pseudormonas aeruginosa)* Biofilm formation and disruption	[134]
MBGs NPs	Cu^2+^	Angiogenesis (synergic effect induced by Si, Ca, and P ions)	In vivo zebrafish model, Subintestinal vessels (SIVs)	[138]
MBG scaffolds	Cu^2+^	Angiogenesis/ Osteostimulation/Antibacterial	In vitro study with hBMSCs (VEGF expression, osteogenic differentiation); *E. coli* viability	[38]
MBG NPs	Sr^2+^	Osteogenesis, Anticlastogenic Anti-inflammatory	Osteoblast-like SAOS, Mesenchymal Stem Cells In vivo studies Murine macrophage cell line	[131,132]
MBG scaffolds	Sr^2+^	Osteogenesis Anticlastogenic	Osteoblast-like cells, MC3T3-E1 (ALP activity, osteogenic expression) In vivo studies	[102,139]
MBG NPs	Ce^3+^	Anti-oxidant, Anti-inflammatory Osteogenic Hemostatic	Mouse fibroblast cells, Osteoblast-like Saos-2 cells In vitro hemostasis assay, platelet adhesion	[137,140]
MBG NPs	Zn^2+^	Osteogenic	Osteoblast-like cells mouse Embryonic fibroblasts cells	[141]
MBG Meso-macroporous 3D scaffolds	Zn^2+^	Antibacterial (also in synergy with antibiotics)	Agar disk diffusion test Planktonic growth inhibition Biofilm degradation	[142]
MBG NPs	Ag^+^	Antibacterial (also in synergy with antibiotics)	Planktonic bacteria model 3D tissue-engineered infected tissue model In vivo infected dental pulp tissue	[136,143]

## References

[1] Vallet-Regí M. (2010). Evolution of Bioceramics within the Field of Biomaterials. Comptes Rendus Chim..

[2] Yanagisawa T., Shimizu T., Kuroda K., Kato C. (1990). The Preparation of Alkyltrimethylammonium-Kanemite Complexes and Their Conversion to Microporous Materials. Bull. Chem. Soc. Jpn..

[3] Kresge C.T., Leonowicz M.E., Roth W.J., Vartuli J.C., Beck J.S. (1992). Ordered Mesoporous Molecular Sieves Synthesized by a Liquid-Crystal Template Mechanism. Nature.

[4] Ying J.Y., Mehnert C.P., Wong M.S. (1999). Synthesis and Applications of Supramolecular-Templated Mesoporous Materials. Angew. Chemie-Int. Ed..

[5] Corma A. (1997). From Microporous to Mesoporous Molecular Sieve Materials and Their Use in Catalysis. Chem. Rev..

[6] Taguchi A., Schüth F. (2005). Ordered Mesoporous Materials in Catalysis. Microporous Mesoporous Mater..

[7] Beck J.S., Vartuli J.C., Roth W.J., Leonowicz M.E., Kresge C.T., Schmitt K.D., Chu C.T.W., Olson D.H., Sheppard E.W., McCullen S.B. (1992). A New Family of Mesoporous Molecular Sieves Prepared with Liquid Crystal Templates. J. Am. Chem. Soc..

[8] Hoffmann F., Cornelius M., Morell J., Fröba M. (2006). Silica-Based Mesoporous Organic-Inorganic Hybrid Materials. Angew. Chemie-Int. Ed..

[9] Yang P., Gai S., Lin J. (2012). Functionalized Mesoporous Silica Materials for Controlled Drug Delivery. Chem. Soc. Rev..

[10] Vallet-Regí M., Rámila A., Del Real R.P., Pérez-Pariente J. (2001). A New Property of MCM-41: Drug Delivery System. Chem. Mater..

[11] Vallet-Regí M., Schüth F., Lozano D., Colilla M., Manzano M. (2022). Engineering Mesoporous Silica Nanoparticles for Drug Delivery: Where Are We after Two Decades?. Chem. Soc. Rev..

[12] Vallet-Regí M. (2022). Our Contributions to Applications of Mesoporous Silica Nanoparticles. Acta Biomater..

[13] Castillo R.R., Lozano D., González B., Manzano M., Izquierdo-Barba I., Vallet-Regí M. (2019). Advances in Mesoporous Silica Nanoparticles for Targeted Stimuli-Responsive Drug Delivery: An Update. Expert Opin. Drug Deliv..

[14] Castillo R.R., Colilla M., Vallet-Regí M. (2017). Advances in Mesoporous Silica-Based Nanocarriers for Co-Delivery and Combination Therapy against Cancer. Expert Opin. Drug Deliv..

[15] Vivero-Escoto J.L., Slowing I.I., Lin V.S.Y., Trewyn B.G. (2010). Mesoporous Silica Nanoparticles for Intracellular Controlled Drug Delivery. Small.

[16] Vallet-Regí M., Balas F., Arcos D. (2007). Mesoporous Materials for Drug Delivery. Angew. Chem.-Int. Ed..

[17] Vallet-Regí M. (2006). Ordered Mesoporous Materials in the Context of Drug Delivery Systems and Bone Tissue Engineering. Chem.-A Eur. J..

[18] Manzano M., Colilla M., Vallet-Regí M. (2009). Drug Delivery from Ordered Mesoporous Matrices. Expert Opin. Drug Deliv..

[19] Zhou S., Zhong Q., Wang Y., Hu P., Zhong W., Huang C.B., Yu Z.Q., Ding C.D., Liu H., Fu J. (2022). Chemically Engineered Mesoporous Silica Nanoparticles-Based Intelligent Delivery Systems for Theranostic Applications in Multiple Cancerous/Non-Cancerous Diseases. Coord. Chem. Rev..

[20] Tella J.O., Adekoya J.A., Ajanaku K.O. (2022). Mesoporous Silica Nanocarriers as Drug Delivery Systems for Anti-Tubercular Agents: A Review. R. Soc. Open Sci..

[21] Álvarez E., González B., Lozano D., Doadrio A.L., Colilla M., Izquierdo-Barba I. (2021). Nanoantibiotics Based in Mesoporous Silica Nanoparticles: New Formulations for Bacterial Infection Treatment. Pharmaceutics.

[22] Manzano M., Vallet-Regí M. (2020). Mesoporous Silica Nanoparticles for Drug Delivery. Adv. Funct. Mater..

[23] Salis A., Fanti M., Medda L., Nairi V., Cugia F., Piludu M., Sogos V., Monduzzi M. (2016). Mesoporous Silica Nanoparticles Functionalized with Hyaluronic Acid and Chitosan Biopolymers. Effect of Functionalization on Cell Internalization. ACS Biomater. Sci. Eng..

[24] Carucci C., Scalas N., Porcheddu A., Piludu M., Monduzzi M., Salis A. (2021). Adsorption and Release of Sulfamethizole from Mesoporous Silica Nanoparticles Functionalised with Triethylenetetramine. Int. J. Mol. Sci..

[25] Castillo R.R., Lozano D., Vallet-Regí M. (2020). Mesoporous Silica Nanoparticles as Carriers for Therapeutic Biomolecules. Pharmaceutics.

[26] Colilla M., Vallet-Regí M. (2020). Targeted Stimuli-Responsive Mesoporous Silica Nanoparticles for Bacterial Infection Treatment. Int. J. Mol. Sci..

[27] Izquierdo-Barba I., Ruiz-González L., Doadrio J.C., González-Calbet J.M., Vallet-Regí M. (2005). Tissue Regeneration: A New Property of Mesoporous Materials. Solid State Sci..

[28] Kokubo T., Kushitani H., Sakka S., Kitsugi T., Yamamuro T. (1990). Solutions Able to Reproduce in Vivo Surface-structure Changes in Bioactive Glass-ceramic A-W3. J. Biomed. Mater. Res..

[29] Vallet-Regí M., Izquierdo-Barba I., Rámila A., Pérez-Pariente J., Babonneau F., González-Calbet J.M. (2005). Phosphorous-Doped MCM-41 as Bioactive Material. Solid State Sci..

[30] Colilla M., Izquierdo-Barba I., Vallet-Regí M. (2010). Phosphorus-Containing SBA-15 Materials as Bisphosphonate Carriers for Osteoporosis Treatment. Microporous Mesoporous Mater..

[31] Horcajada P., Rámila A., Boulahya K., González-Calbet J., Vallet-Regí M. (2004). Bioactivity in Ordered Mesoporous Materials. Solid State Sci..

[32] Vallet-Regí M., Ruiz-González L., Izquierdo-Barba I., González-Calbet J.M. (2006). Revisiting Silica Based Ordered Mesoporous Materials: Medical Applications. J. Mater. Chem..

[33] Vallet-Regí M. (2010). Nanostructured Mesoporous Silica Matrices in Nanomedicine. J. Intern. Med..

[34] Izquierdo-Barba I., Vallet-Regí M. (2011). Fascinating Properties of Bioactive Templated Glasses: A New Generation of Nanostructured Bioceramics. Solid State Sci..

[35] Vallet-Regí M., Izquierdo-Barba I., Colilla M. (2012). Structure and Functionalization of Mesoporous Bioceramics for Bone Tissue Regeneration and Local Drug Delivery. Philos. Trans. R. Soc. A Math. Phys. Eng. Sci..

[36] Salinas A.J., Esbrit P. (2022). Mesoporous Bioglasses Enriched with Bioactive Agents for Bone Repair, with a Special Highlight of María Vallet-Regí’s Contribution. Pharmaceutics.

[37] Izquierdo-Barba I., Vallet-Regí M. (2015). Mesoporous Bioactive Glasses: Relevance of Their Porous Structure Compared to That of Classical Bioglasses. Biomed. Glas..

[38] Wu C., Zhou Y., Xu M., Han P., Chen L., Chang J., Xiao Y. (2013). Copper-Containing Mesoporous Bioactive Glass Scaffolds with Multifunctional Properties of Angiogenesis Capacity, Osteostimulation and Antibacterial Activity. Biomaterials.

[39] Arcos D., Vallet-Regí M. (2010). Sol–Gel Silica-Based Biomaterials and Bone Tissue Regeneration. Acta Biomater..

[40] Izquierdo-Barba I., Arcos D., Sakamoto Y., Terasaki O., López-Noriega A., Vallet-Regí M. (2008). High-Performance Mesoporous Bioceramics Mimicking Bone Mineralization. Chem. Mater..

[41] Vallet-Regí M., Salinas A.J. (2021). Mesoporous Bioactive Glasses for Regenerative Medicine. Mater. Today Bio.

[42] Arcos D., Izquierdo-Barba I., Vallet-Regí M. (2009). Promising Trends of Bioceramics in the Biomaterials Field. J. Mater. Sci. Mater. Med..

[43] Yan X., Yu C., Zhou X., Tang J., Zhao D. (2004). Highly Ordered Mesoporous Bioactive Glasses with Superior in Vitro Bone-Forming Bioactivities. Angew. Chemie-Int. Ed..

[44] López-Noriega A., Arcos D., Izquierdo-Barba I., Sakamoto Y., Terasaki O., Vallet-Regí M. (2006). Ordered Mesoporous Bioactive Glasses for Bone Tissue Regeneration. Chem. Mater..

[45] Brinker C.J., Lu Y., Sellinger A., Fan H. (1999). Evaporation-Induced Self-Assembly: Nanostructures Made Easy. Adv. Mater..

[46] Yan X.X., Deng H.X., Huang X.H., Lu G.Q., Qiao S.Z., Zhao D.Y., Yu C.Z. (2005). Mesoporous Bioactive Glasses. I. Synthesis and Structural Characterization. J. Non. Cryst. Solids.

[47] Yan X., Huang X., Yu C., Deng H., Wang Y., Zhang Z., Qiao S., Lu G., Zhao D. (2006). The In-Vitro Bioactivity of Mesoporous Bioactive Glasses. Biomaterials.

[48] Elgayar I., Aliev A.E., Boccaccini A.R., Hill R.G. (2005). Structural Analysis of Bioactive Glasses. J. Non. Cryst. Solids.

[49] Vallet-Regí M., Salinas A.J., Ramírez-Castellanos J., González-Calbet J.M. (2005). Nanostructure of Bioactive Sol-Gel Glasses and Organic-Inorganic Hybrids. Chem. Mater..

[50] Tilocca A. (2009). Structural Models of Bioactive Glasses from Molecular Dynamics Simulations. Proc. R. Soc. A Math. Phys. Eng. Sci..

[51] Leonova E., Izquierdo-Barba I., Arcos D., López-Noriega A., Hedin N., Vallet-Regí M., Edén M. (2008). Multinuclear Solid-State NMR Studies of Ordered Mesoporous Bioactive Glasses. J. Phys. Chem. C.

[52] Mathew R., Turdean-Ionescu C., Yu Y., Stevensson B., Izquierdo-Barba I., García A., Arcos D., Vallet-Regí M., Edén M. (2017). Proton Environments in Biomimetic Calcium Phosphates Formed from Mesoporous Bioactive CaO-SiO_2_-P_2_O_5_ Glasses in Vitro: Insights from Solid-State NMR. J. Phys. Chem. C.

[53] Mathew R., Turdean-Ionescu C., Stevensson B., Izquierdo-Barba I., García A., Arcos D., Vallet-Regí M., Edén M. (2013). Direct Probing of the Phosphate-Ion Distribution in Bioactive Silicate Glasses by Solid-State NMR: Evidence for Transitions between Random/Clustered Scenarios. Chem. Mater..

[54] Gunawidjaja P.N., Mathew R., Lo A.Y.H., Izquierdo-Barba I., García A., Arcos D., Vallet-Regí M., Edén M. (2012). Local Structures of Mesoporous Bioactive Glasses and Their Surface Alterations in Vitro: Inferences from Solid-State Nuclear Magnetic Resonance. Philos. Trans. R. Soc. A Math. Phys. Eng. Sci..

[55] García A., Cicuéndez M., Izquierdo-Barba I., Arcos D., Vallet-Regí M. (2009). Essential Role of Calcium Phosphate Heterogeneities in 2D-Hexagonal and 3D-Cubic SiO_2_-CaO-P_2_O_5_ Mesoporous Bioactive Glasses. Chem. Mater..

[56] Misra S.K., Mohn D., Brunner T.J., Stark W.J., Philip S.E., Roy I., Salih V., Knowles J.C., Boccaccini A.R. (2008). Comparison of Nanoscale and Microscale Bioactive Glass on the Properties of P(3HB)/Bioglass^®^ Composites. Biomaterials.

[57] Liang Q., Hu Q., Miao G., Yuan B., Chen X. (2015). A Facile Synthesis of Novel Mesoporous Bioactive Glass Nanoparticles with Various Morphologies and Tunable Mesostructure by Sacrificial Liquid Template Method. Mater. Lett..

[58] Hong B.-J., Hsiao C.-W., Bakare F., Sun J.-T., Shih S.-J. (2018). Effect of Acetic Acid Concentration on Pore Structure for Mesoporous Bioactive Glass during Spray Pyrolysis. Materials.

[59] Peng T.-Y., Tsai P.-Y., Chen M.-S., Mine Y., Wu S.-H., Chen C.-Y., Lin D.-J., Lin C.-K. (2021). Mesoporous Properties of Bioactive Glass Synthesized by Spray Pyrolysis with Various Polyethylene Glycol and Acid Additions. Polymers.

[60] Shi Q., Wang J., Zhang J., Fan J., Stucky G.D. (2006). Rapid-Setting, Mesoporous, Bioactive Glass Cements That Induce Accelerated in Vitro Apatite Formation. Adv. Mater..

[61] De Cremer K., Braem A., Gerits E., De Brucker K., Vandamme K., Martens J.A., Michiels J., Vleugels J., Cammue B.P.A., Thevissen K. (2017). Controlled Release of Chlorhexidine from a Mesoporous Silica-Containing Macroporous Titanium Dental Implant Prevents Microbial Biofilm Formation. Eur. Cells Mater..

[62] Baino F., Fiorilli S., Vitale-Brovarone C. (2017). Composite Biomaterials Based on Sol-Gel Mesoporous Silicate Glasses: A Review. Bioengineering.

[63] Baino F., Fiume E. (2020). 3D Printing of Hierarchical Scaffolds Based on Mesoporous Bioactive Glasses (MBGs)—Fundamentals and Applications. Materials.

[64] Gupta S., Majumdar S., Krishnamurthy S. (2021). Bioactive Glass: A Multifunctional Delivery System. J. Control. Release.

[65] Kong C.H., Steffi C., Shi Z., Wang W. (2018). Development of Mesoporous Bioactive Glass Nanoparticles and Its Use in Bone Tissue Engineering. J. Biomed. Mater. Res.-Part B Appl. Biomater..

[66] Lalzawmliana V., Anand A., Roy M., Kundu B., Nandi S.K. (2020). Mesoporous Bioactive Glasses for Bone Healing and Biomolecules Delivery. Mater. Sci. Eng. C.

[67] Zheng K., Kang J., Rutkowski B., Gawȩda M., Zhang J., Wang Y., Founier N., Sitarz M., Taccardi N., Boccaccini A.R. (2019). Toward Highly Dispersed Mesoporous Bioactive Glass Nanoparticles with High Cu Concentration Using Cu/Ascorbic Acid Complex as Precursor. Front. Chem..

[68] Jiménez-Holguín J., Sánchez-Salcedo S., Cicuéndez M., Vallet-Regí M., Salinas A.J. (2022). Cu-Doped Hollow Bioactive Glass Nanoparticles for Bone Infection Treatment. Pharmaceutics.

[69] Wu C., Fan W., Chang J. (2013). Functional Mesoporous Bioactive Glass Nanospheres: Synthesis, High Loading Efficiency, Controllable Delivery of Doxorubicin and Inhibitory Effect on Bone Cancer Cells. J. Mater. Chem. B.

[70] Dey N., Santhiya D., Das A. (2022). Bio-Inspired Synthesis of Hollow Mesoporous Bioactive Glass Nanoparticles Using Calcium Carbonate as Solid Template. ChemistrySelect.

[71] Lin H.-N., Peng T.-Y., Kung Y.-R., Chiou Y.-J., Chang W.-M., Wu S.-H., Mine Y., Chen C.-Y., Lin C.-K. (2022). Effects of the Methyl Methacrylate Addition, Polymerization Temperature and Time on the MBG@PMMA Core-Shell Structure and Its Application as Addition in Electrospun Composite Fiber Bioscaffold. Ceram. Int..

[72] Wu C., Chang J. (2012). Mesoporous Bioactive Glasses: Structure Characteristics, Drug/Growth Factor Delivery and Bone Regeneration Application. Interface Focus.

[73] Salinas A.J., Shruti S., Malavasi G., Menabue L., Vallet-Regí M. (2011). Substitutions of Cerium, Gallium and Zinc in Ordered Mesoporous Bioactive Glasses. Acta Biomater..

[74] Shruti S., Salinas A.J., Lusvardi G., Malavasi G., Menabue L., Vallet-Regí M. (2013). Mesoporous Bioactive Scaffolds Prepared with Cerium-, Gallium- and Zinc-Containing Glasses. Acta Biomater..

[75] El-Fiqi A., Kim H.W. (2021). Iron Ions-Releasing Mesoporous Bioactive Glass Ultrasmall Nanoparticles Designed as Ferroptosis-Based Bone Cancer Nanotherapeutics: Ultrasonic-Coupled Sol–Gel Synthesis, Properties and Iron Ions Release. Mater. Lett..

[76] Wu C., Chang J. (2014). Multifunctional Mesoporous Bioactive Glasses for Effective Delivery of Therapeutic Ions and Drug/Growth Factors. J. Control. Release.

[77] Hoppe A., Güldal N.S., Boccaccini A.R. (2011). A Review of the Biological Response to Ionic Dissolution Products from Bioactive Glasses and Glass-Ceramics. Biomaterials.

[78] Zhu H., Zheng K., Boccaccini A.R. (2021). Multi-Functional Silica-Based Mesoporous Materials for Simultaneous Delivery of Biologically Active Ions and Therapeutic Biomolecules. Acta Biomater..

[79] Sharifi E., Bigham A., Yousefiasl S., Trovato M., Ghomi M., Esmaeili Y., Samadi P., Zarrabi A., Ashrafizadeh M., Sharifi S. (2022). Mesoporous Bioactive Glasses in Cancer Diagnosis and Therapy: Stimuli-Responsive, Toxicity, Immunogenicity, and Clinical Translation. Adv. Sci..

[80] Vallet-Regí M., Lozano D., González B., Izquierdo-Barba I. (2020). Biomaterials against Bone Infection. Adv. Healthc. Mater..

[81] Izquierdo-Barba I., Colilla M., Vallet-Regí M. (2016). Zwitterionic Ceramics for Biomedical Applications. Acta Biomater..

[82] Turnbull G., Clarke J., Picard F., Riches P., Jia L., Han F., Li B., Shu W. (2018). 3D Bioactive Composite Scaffolds for Bone Tissue Engineering. Bioact. Mater..

[83] Hollister S.J. (2009). Scaffold Design and Manufacturing: From Concept to Clinic. Adv. Mater..

[84] Loh Q.L., Choong C. (2013). Three-Dimensional Scaffolds for Tissue Engineering Applications: Role of Porosity and Pore Size. Tissue Eng.-Part B Rev..

[85] Salinas A.J., Esbrit P., Vallet-Regí M. (2013). A Tissue Engineering Approach Based on the Use of Bioceramics for Bone Repair. Biomater. Sci..

[86] García A., Cabañas M.V., Peña J., Sánchez-Salcedo S. (2021). Design of 3D Scaffolds for Hard Tissue Engineering: From Apatites to Silicon Mesoporous Materials. Pharmaceutics.

[87] Wegst U.G.K., Bai H., Saiz E., Tomsia A.P., Ritchie R.O. (2015). Bioinspired Structural Materials. Nat. Mater..

[88] Koester K.J., Ager J.W., Ritchie R.O. (2008). The True Toughness of Human Cortical Bone Measured with Realistically Short Cracks. Nat. Mater..

[89] Yun H.S., Kim S.E., Hyun Y.T., Heo S.J., Shin J.W. (2008). Hierarchically Mesoporous-Macroporous Bioactive Glasses Scaffolds for Bone Tissue Regeneration. J. Biomed. Mater. Res.-Part B Appl. Biomater..

[90] Lian R., Xie P., Xiao L., Iqbal Z., Zhang S., Kohn J., Qu X., Liu C., Li Y. (2021). Rational Design and Fabrication of Biomimetic Hierarchical Scaffolds With Bone-Matchable Strength for Bone Regeneration. Front. Mater..

[91] Nikolova M.P., Chavali M.S. (2019). Recent Advances in Biomaterials for 3D Scaffolds: A Review. Bioact. Mater..

[92] Wu C., Miron R., Sculean A., Kaskel S., Doert T., Schulze R., Zhang Y. (2011). Proliferation, Differentiation and Gene Expression of Osteoblasts in Boron-Containing Associated with Dexamethasone Deliver from Mesoporous Bioactive Glass Scaffolds. Biomaterials.

[93] Tang W., Lin D., Yu Y., Niu H., Guo H., Yuan Y., Liu C. (2016). Bioinspired Trimodal Macro/Micro/Nano-Porous Scaffolds Loading RhBMP-2 for Complete Regeneration of Critical Size Bone Defect. Acta Biomater..

[94] Ciraldo F.E., Arango-Ospina M., Goldmann W.H., Beltrán A.M., Detsch R., Gruenewald A., Roether J.A., Boccaccini A.R. (2021). Fabrication and Characterization of Ag- and Ga-Doped Mesoporous Glass-Coated Scaffolds Based on Natural Marine Sponges with Improved Mechanical Properties. J. Biomed. Mater. Res.-Part A.

[95] Wang X., Ruan J.M., Chen Q.Y. (2009). Effects of Surfactants on the Microstructure of Porous Ceramic Scaffolds Fabricated by Foaming for Bone Tissue Engineering. Mater. Res. Bull..

[96] Zadpoor A.A., Malda J. (2017). Additive Manufacturing of Biomaterials, Tissues, and Organs. Ann. Biomed. Eng..

[97] Liaw C.Y., Guvendiren M. (2017). Current and Emerging Applications of 3D Printing in Medicine. Biofabrication.

[98] Mirkhalaf M., Men Y., Wang R., No Y., Zreiqat H. (2022). Personalized 3D Printed Bone Scaffolds: A Review. Acta Biomater..

[99] Zhang L., Yang G., Johnson B.N., Jia X. (2019). Three-Dimensional (3D) Printed Scaffold and Material Selection for Bone Repair. Acta Biomater..

[100] García A., Izquierdo-Barba I., Colilla M., De Laorden C.L., Vallet-Regí M. (2011). Preparation of 3-D Scaffolds in the SiO_2_-P_2_O_5_ System with Tailored Hierarchical Meso-Macroporosity. Acta Biomater..

[101] Wu C., Luo Y., Cuniberti G., Xiao Y., Gelinsky M. (2011). Three-Dimensional Printing of Hierarchical and Tough Mesoporous Bioactive Glass Scaffolds with a Controllable Pore Architecture, Excellent Mechanical Strength and Mineralization Ability. Acta Biomater..

[102] Zhang J., Zhao S., Zhu Y., Huang Y., Zhu M., Tao C., Zhang C. (2014). Three-Dimensional Printing of Strontium-Containing Mesoporous Bioactive Glass Scaffolds for Bone Regeneration. Acta Biomater..

[103] Zhao S., Zhang J., Zhu M., Zhang Y., Liu Z., Tao C., Zhu Y., Zhang C. (2015). Three-Dimensional Printed Strontium-Containing Mesoporous Bioactive Glass Scaffolds for Repairing Rat Critical-Sized Calvarial Defects. Acta Biomater..

[104] Qi X., Pei P., Zhu M., Du X., Xin C., Zhao S., Li X., Zhu Y. (2017). Three Dimensional Printing of Calcium Sulfate and Mesoporous Bioactive Glass Scaffolds for Improving Bone Regeneration in Vitro and in Vivo. Sci. Rep..

[105] Luo Y., Wu C., Lode A., Gelinsky M. (2013). Hierarchical Mesoporous Bioactive Glass/Alginate Composite Scaffolds Fabricated by Three-Dimensional Plotting for Bone Tissue Engineering. Biofabrication.

[106] Yun H.S., Kim S.E., Park E.K. (2011). Bioactive Glass-Poly (ε-Caprolactone) Composite Scaffolds with 3 Dimensionally Hierarchical Pore Networks. Mater. Sci. Eng. C.

[107] Min Z., Kun L., Yufang Z., Jianhua Z., Xiaojian Y. (2015). 3D-Printed Hierarchical Scaffold for Localized Isoniazid/Rifampin Drug Delivery and Osteoarticular Tuberculosis Therapy. Acta Biomater..

[108] Sánchez-Salcedo S., García A., González-Jiménez A., Vallet-Regí M. (2022). Antibacterial Effect of 3D Printed Mesoporous Bioactive Glass Scaffolds Doped with Metallic Silver Nanoparticles. Acta Biomater..

[109] Cicuéndez M., Doadrio J.C., Hernández A., Portolés M.T., Izquierdo-Barba I., Vallet-Regí M. (2018). Multifunctional PH Sensitive 3D Scaffolds for Treatment and Prevention of Bone Infection. Acta Biomater..

[110] Cicuéndez M., Portolés P., Montes-Casado M., Izquierdo-Barba I., Vallet-Regí M., Portolés M.T. (2014). Effects of 3D Nanocomposite Bioceramic Scaffolds on the Immune Response. J. Mater. Chem. B.

[111] Wang Z., Lin D., Wang M., Mao R., Zhao H., Huang X., GF Shen S. (2022). Seamless Route of Self-Assembly and 3D Printing to Fabricate Hierarchical Mesoporous Bioactive Glass Scaffold for Customized Bone Regeneration with Enhanced Efficacy. Chem. Eng. J..

[112] Heras C., Sanchez-Salcedo S., Lozano D., Peña J., Esbrit P., Vallet-Regí M., Salinas A.J. (2019). Osteostatin Potentiates the Bioactivity of Mesoporous Glass Scaffolds Containing Zn2+ Ions in Human Mesenchymal Stem Cells. Acta Biomater..

[113] Gómez-Cerezo N., Sánchez-Salcedo S., Izquierdo-Barba I., Arcos D., Vallet-Regí M. (2016). In Vitro Colonization of Stratified Bioactive Scaffolds by Pre-Osteoblast Cells. Acta Biomater..

[114] Gómez-Cerezo N., Casarrubios L., Saiz-Pardo M., Ortega L., de Pablo D., Díaz-Güemes I., Fernández-Tomé B., Enciso S., Sánchez-Margallo F.M., Portolés M.T. (2019). Mesoporous Bioactive Glass/ε-Polycaprolactone Scaffolds Promote Bone Regeneration in Osteoporotic Sheep. Acta Biomater..

[115] Gómez-Cerezo M.N., Peña J., Ivanovski S., Arcos D., Vallet-Regí M., Vaquette C. (2021). Multiscale Porosity in Mesoporous Bioglass 3D-Printed Scaffolds for Bone Regeneration. Mater. Sci. Eng. C.

[116] Lozano D., Gil-Albarova J., Heras C., Sánchez-Salcedo S., Gómez-Palacio V.E., Gómez-Blasco A., Doadrio J.C., Vallet-Regí M., Salinas A.J. (2020). ZnO-Mesoporous Glass Scaffolds Loaded with Osteostatin and Mesenchymal Cells Improve Bone Healing in a Rabbit Bone Defect. J. Mater. Sci. Mater. Med..

[117] García-Alvarez R., Izquierdo-Barba I., Vallet-Regí M. (2017). 3D Scaffold with Effective Multidrug Sequential Release against Bacteria Biofilm. Acta Biomater..

[118] Jiménez-Holguín J., López-Hidalgo A., Sánchez-Salcedo S., Peña J., Vallet-Regí M., Salinas A.J. (2020). Strontium-Modified Scaffolds Based on Mesoporous Bioactive Glasses/Polyvinyl Alcohol Composites for Bone Regeneration. Materials.

[119] Saberi A., Behnamghader A., Aghabarari B., Yousefi A., Majda D., Huerta M.V.M., Mozafari M. (2022). 3D Direct Printing of Composite Bone Scaffolds Containing Polylactic Acid and Spray Dried Mesoporous Bioactive Glass-Ceramic Microparticles. Int. J. Biol. Macromol..

[120] Fu S., Du X., Zhu M., Tian Z., Wei D., Zhu Y. (2019). 3D Printing of Layered Mesoporous Bioactive Glass/Sodium Alginatesodium Alginate Scaffolds with Controllable Dual-Drug Release Behaviors. Biomed. Mater..

[121] Du X., Wei D., Huang L., Zhu M., Zhang Y., Zhu Y. (2019). 3D Printing of Mesoporous Bioactive Glass/Silk Fibroin Composite Scaffolds for Bone Tissue Engineering. Mater. Sci. Eng. C.

[122] Ferreira A.M., Gentile P., Chiono V., Ciardelli G. (2012). Collagen for Bone Tissue Regeneration. Acta Biomater..

[123] Montalbano G., Borciani G., Cerqueni G., Licini C., Banche-Niclot F., Janner D., Sola S., Fiorilli S., Mattioli-Belmonte M., Ciapetti G. (2020). Collagen Hybrid Formulations for the 3d Printing of Nanostructured Bone Scaffolds: An Optimized Genipin-Crosslinking Strategy. Nanomaterials.

[124] Gaihre B., Uswatta S., Jayasuriya A. (2017). Reconstruction of Craniomaxillofacial Bone Defects Using Tissue-Engineering Strategies with Injectable and Non-Injectable Scaffolds. J. Funct. Biomater..

[125] Dreifke M.B., Ebraheim N.A., Jayasuriya A.C. (2013). Investigation of Potential Injectable Polymeric Biomaterials for Bone Regeneration. J. Biomed. Mater. Res.-Part A.

[126] Dimatteo R., Darling N.J., Segura T. (2018). In Situ Forming Injectable Hydrogels for Drug Delivery and Wound Repair. Adv. Drug Deliv. Rev..

[127] Giannini G., Mauro V., Agostino T., Gianfranco B. (2004). Use of Autologous Fibrin-Platelet Glue and Bone Fragments in Maxillofacial Surgery. Transfus. Apher. Sci..

[128] Noori A., Ashrafi S.J., Vaez-Ghaemi R., Hatamian-Zaremi A., Webster T.J. (2017). A Review of Fibrin and Fibrin Composites for Bone Tissue Engineering. Int. J. Nanomed..

[129] Zhou L., Fan L., Zhang F.M., Jiang Y., Cai M., Dai C., Luo Y.A., Tu L.J., Zhou Z.N., Li X.J. (2021). Hybrid Gelatin/Oxidized Chondroitin Sulfate Hydrogels Incorporating Bioactive Glass Nanoparticles with Enhanced Mechanical Properties, Mineralization, and Osteogenic Differentiation. Bioact. Mater..

[130] Zhao H., Wang X., Jin A., Wang M., Wang Z., Huang X., Dai J., Wang X., Lin D., Shen S.G. (2022). Reducing Relapse and Accelerating Osteogenesis in Rapid Maxillary Expansion Using an Injectable Mesoporous Bioactive Glass/Fibrin Glue Composite Hydrogel. Bioact. Mater..

[131] Fiorilli S., Molino G., Pontremoli C., Iviglia G., Torre E., Cassinelli C., Morra M., Vitale-Brovarone C. (2018). The Incorporation of Strontium to Improve Bone-Regeneration Ability of Mesoporous Bioactive Glasses. Materials.

[132] Lee J.H., Mandakhbayar N., El-Fiqi A., Kim H.W. (2017). Intracellular Co-Delivery of Sr Ion and Phenamil Drug through Mesoporous Bioglass Nanocarriers Synergizes BMP Signaling and Tissue Mineralization. Acta Biomater..

[133] Bari A., Bloise N., Fiorilli S., Novajra G., Vallet-Regí M., Bruni G., Torres-Pardo A., González-Calbet J.M., Visai L., Vitale-Brovarone C. (2017). Copper-Containing Mesoporous Bioactive Glass Nanoparticles as Multifunctional Agent for Bone Regeneration. Acta Biomater..

[134] Paterson T.E., Bari A., Bullock A.J., Turner R., Montalbano G., Fiorilli S., Vitale-Brovarone C., MacNeil S., Shepherd J. (2020). Multifunctional Copper-Containing Mesoporous Glass Nanoparticles as Antibacterial and Proangiogenic Agents for Chronic Wounds. Front. Bioeng. Biotechnol..

[135] El-Fiqi A., Mandakhbayar N., Jo S.B., Knowles J.C., Lee J.H., Kim H.W. (2021). Nanotherapeutics for Regeneration of Degenerated Tissue Infected by Bacteria through the Multiple Delivery of Bioactive Ions and Growth Factor with Antibacterial/Angiogenic and Osteogenic/Odontogenic Capacity. Bioact. Mater..

[136] Zheng K., Balasubramanian P., Paterson T.E., Stein R., MacNeil S., Fiorilli S., Vitale-Brovarone C., Shepherd J., Boccaccini A.R. (2019). Ag Modified Mesoporous Bioactive Glass Nanoparticles for Enhanced Antibacterial Activity in 3D Infected Skin Model. Mater. Sci. Eng. C.

[137] Zheng K., Torre E., Bari A., Taccardi N., Cassinelli C., Morra M., Fiorilli S., Vitale-Brovarone C., Iviglia G., Boccaccini A.R. (2020). Antioxidant Mesoporous Ce-Doped Bioactive Glass Nanoparticles with Anti-Inflammatory and pro-Osteogenic Activities. Mater. Today Bio.

[138] Romero-Sánchez L.B., Marí-Beffa M., Carrillo P., Medina M.Á., Díaz-Cuenca A. (2018). Copper-Containing Mesoporous Bioactive Glass Promotes Angiogenesis in an in Vivo Zebrafish Model. Acta Biomater..

[139] Zhang Y., Wei L., Chang J., Miron R.J., Shi B., Yi S., Wu C. (2013). Strontium-Incorporated Mesoporous Bioactive Glass Scaffolds Stimulating in Vitro Proliferation and Differentiation of Bone Marrow Stromal Cells and in Vivo Regeneration of Osteoporotic Bone Defects. J. Mater. Chem. B.

[140] Liu J., Zhou X., Zhang Y., Wang A., Zhu W., Xu M., Zhuang S. (2022). Rapid Hemostasis and High Bioactivity Cerium-Containing Mesoporous Bioglass for Hemostatic Materials. J. Biomed. Mater. Res.-Part B Appl. Biomater..

[141] Neščáková Z., Zheng K., Liverani L., Nawaz Q., Galusková D., Kaňková H., Michálek M., Galusek D., Boccaccini A.R. (2019). Multifunctional Zinc Ion Doped Sol-Gel Derived Mesoporous Bioactive Glass Nanoparticles for Biomedical Applications. Bioact. Mater..

[142] Heras C., Jiménez-Holguín J., Doadrio A.L., Vallet-Regí M., Sánchez-Salcedo S., Salinas A.J. (2020). Multifunctional Antibiotic- and Zinc-Containing Mesoporous Bioactive Glass Scaffolds to Fight Bone Infection. Acta Biomater..

[143] Lee J.H., El-Fiqi A., Mandakhbayar N., Lee H.H., Kim H.W. (2017). Drug/Ion Co-Delivery Multi-Functional Nanocarrier to Regenerate Infected Tissue Defect. Biomaterials.

[144] Campoccia D., Montanaro L., Arciola C.R. (2013). A Review of the Biomaterials Technologies for Infection-Resistant Surfaces. Biomaterials.

[145] Colilla M., Izquierdo-Barba I., Vallet-Regí M. (2018). The Role of Zwitterionic Materials in the Fight against Proteins and Bacteria. Medicines.

[146] Cheng G., Zhang Z., Chen S., Bryers J.D., Jiang S. (2007). Inhibition of Bacterial Adhesion and Biofilm Formation on Zwitterionic Surfaces. Biomaterials.

[147] Jiang S., Cao Z. (2010). Ultralow-Fouling, Functionalizable, and Hydrolyzable Zwitterionic Materials and Their Derivatives for Biological Applications. Adv. Mater..

[148] Chen S., Li L., Zhao C., Zheng J. (2010). Surface Hydration: Principles and Applications toward Low-Fouling/Nonfouling Biomaterials. Polymer.

[149] Rosen J.E., Gu F.X. (2011). Surface Functionalization of Silica Nanoparticles with Cysteine: A Low-Fouling Zwitterionic Surface. Langmuir.

[150] Villegas M.F., Garcia-Uriostegui L., Rodríguez O., Izquierdo-Barba I., Salinas A.J., Toriz G., Vallet-Regí M., Delgado E. (2017). Lysine-Grafted MCM-41 Silica as an Antibacterial Biomaterial. Bioengineering.

[151] Izquierdo-Barba I., Sánchez-Salcedo S., Colilla M., Feito M.J., Ramírez-Santillán C., Portolés M.T., Vallet-Regí M. (2011). Inhibition of Bacterial Adhesion on Biocompatible Zwitterionic SBA-15 Mesoporous Materials. Acta Biomater..

[152] Han L., Ruan J., Li Y., Terasaki O., Che S. (2007). Synthesis and Characterization of the Amphoteric Amino Acid Bifunctional Mesoporous Silica. Chem. Mater..

[153] Colilla M., Izquierdo-Barba I., Sánchez-Salcedo S., Fierro J.L.G., Hueso J.L., Vallet-Regí M. (2010). Synthesis and Characterization of Zwitterionic SBA-15 Nanostructured Materials. Chem. Mater..

[154] Sánchez-Salcedo S., Colilla M., Izquierdo-Barba I., Vallet-Regí M. (2013). Design and Preparation of Biocompatible Zwitterionic Hydroxyapatite. J. Mater. Chem. B.

[155] Rodríguez-Palomo A., Monopoli D., Afonso H., Izquierdo-Barba I., Vallet-Regí M. (2016). Surface Zwitterionization of Customized 3D Ti6Al4V Scaffolds: A Promising Alternative to Eradicate Bone Infection. J. Mater. Chem. B.

[156] Sánchez-Salcedo S., Colilla M., Izquierdo-Barba I., Vallet-Regí M. (2016). Preventing Bacterial Adhesion on Scaffolds for Bone Tissue Engineering. Int. J. Bioprinting.

[157] Encinas N., Angulo M., Astorga C., Colilla M., Izquierdo-Barba I., Vallet-Regí M. (2019). Mixed-Charge Pseudo-Zwitterionic Mesoporous Silica Nanoparticles with Low-Fouling and Reduced Cell Uptake Properties. Acta Biomater..

[158] Sánchez-Salcedo S., García A., Vallet-Regí M. (2017). Prevention of Bacterial Adhesion to Zwitterionic Biocompatible Mesoporous Glasses. Acta Biomater..

[159] Pontremoli C., Izquierdo-Barba I., Montalbano G., Vallet-Regí M., Vitale-Brovarone C., Fiorilli S. (2020). Strontium-Releasing Mesoporous Bioactive Glasses with Anti-Adhesive Zwitterionic Surface as Advanced Biomaterials for Bone Tissue Regeneration. J. Colloid Interface Sci..

[160] Naruphontjirakul P., Tsigkou O., Li S., Porter A.E., Jones J.R. (2019). Human Mesenchymal Stem Cells Differentiate into an Osteogenic Lineage in Presence of Strontium Containing Bioactive Glass Nanoparticles. Acta Biomater..

[161] El-Fiqi A., Kim J.H., Kim H.W. (2015). Osteoinductive Fibrous Scaffolds of Biopolymer/Mesoporous Bioactive Glass Nanocarriers with Excellent Bioactivity and Long-Term Delivery of Osteogenic Drug. ACS Appl. Mater. Interfaces.

[162] Reginster J.Y.L. (1993). Ipriflavone: Pharmacological Properties and Usefulness in Postmenopausal Osteoporosis. Bone Miner..

[163] Kim S.H., Lee M.G. (2002). Pharmacokinetics of Ipriflavone, an Isoflavone Derivative, after Intravenous and Oral Administration to Rats: Hepatic and Intestinal First-Pass Effects. Life Sci..

[164] O’Connell M.B. (1995). Pharmacokinetic and Pharmacologic Variation Between Different Estrogen Products. J. Clin. Pharmacol..

[165] López-Noriega A., Arcos D., Vallet-Regí M. (2010). Functionalizing Mesoporous Bioglasses for Long-Term Anti-Osteoporotic Drug Delivery. Chem.-Eur. J..

[166] Casarrubios L., Gómez-Cerezo N., Feito M.J., Vallet-Regí M., Arcos D., Portolés M.T. (2018). Incorporation and Effects of Mesoporous SiO_2_-CaO Nanospheres Loaded with Ipriflavone on Osteoblast/Osteoclast Cocultures. Eur. J. Pharm. Biopharm..

[167] Mathew R., Gunawidjaja P.N., Izquierdo-Barba I., Jansson K., García A., Arcos D., Vallet-Regí M., Edén M. (2011). Solid-State 31P and 1H NMR Investigations of Amorphous and Crystalline Calcium Phosphates Grown Biomimetically from a Mesoporous Bioactive Glass. J. Phys. Chem. C.

[168] Gómez-Cerezo N., Izquierdo-Barba I., Arcos D., Vallet-Regí M. (2015). Tailoring the Biological Response of Mesoporous Bioactive Materials. J. Mater. Chem. B.

[169] Casarrubios L., Polo-Montalvo A., Serrano M.C., Feito M.J., Vallet-Regí M., Arcos D., Portolés M.T. (2021). Effects of Ipriflavone-Loaded Mesoporous Nanospheres on the Differentiation of Endothelial Progenitor Cells and Their Modulation by Macrophages. Nanomaterials.

[170] Wang D., Steffi C., Wang Z., Kong C.H., Lim P.N., Shi Z., Thian E.S., Wang W. (2018). Beta-Cyclodextrin Modified Mesoporous Bioactive Glass Nanoparticles/Silk Fibroin Hybrid Nanofibers as an Implantable Estradiol Delivery System for the Potential Treatment of Osteoporosis. Nanoscale.

[171] Ezra A., Golomb G. (2000). Administration Routes and Delivery Systems of Bisphosphonates for the Treatment of Bone Resorption. Adv. Drug Deliv. Rev..

[172] Wang X., Zeng D., Weng W., Huang Q., Zhang X., Wen J., Wu J., Jiang X. (2018). Alendronate Delivery on Amino Modified Mesoporous Bioactive Glass Scaffolds to Enhance Bone Regeneration in Osteoporosis Rats. Artif. Cells Nanomed. Biotechnol..

[173] Liu L., Zhao F., Chen X., Luo M., Yang Z., Cao X., Miao G., Chen D., Chen X. (2020). Local Delivery of FTY720 in Mesoporous Bioactive Glass Improves Bone Regeneration by Synergistically Immunomodulating Osteogenesis and Osteoclastogenesis. J. Mater. Chem. B.

[174] Jia M., Nie Y., Cao D.P., Xue Y.Y., Wang J.S., Zhao L., Rahman K., Zhang Q.Y., Qin L.P. (2012). Potential Antiosteoporotic Agents from Plants: A Comprehensive Review. Evid.-Based Complement. Altern. Med..

[175] Wang Z., Wang D., Yang D., Zhen W., Zhang J., Peng S. (2018). The Effect of Icariin on Bone Metabolism and Its Potential Clinical Application. Osteoporos. Int..

[176] Mosqueira L., Barrioni B.R., Martins T., Ocarino N.D.M., Serakides R., Pereira M.D.M. (2020). In Vitro Effects of the Co-Release of Icariin and Strontium from Bioactive Glass Submicron Spheres on the Reduced Osteogenic Potential of Rat Osteoporotic Bone Marrow Mesenchymal Stem Cells. Biomed. Mater..

[177] Shen X., Yu P., Chen H., Wang J., Lu B., Cai X., Gu C., Liang G., Hao D., Ma Q. (2020). Icariin Controlled Release on a Silk Fibroin/Mesoporous Bioactive Glass Nanoparticles Scaffold for Promoting Stem Cell Osteogenic Differentiation. RSC Adv..

[178] Park K.W., Waki H., Kim W.-K., Davies B.S.J., Young S.G., Parhami F., Tontonoz P. (2009). The Small Molecule Phenamil Induces Osteoblast Differentiation and Mineralization. Mol. Cell. Biol..

[179] Lo K.W.H., Jiang T., Gagnon K.A., Nelson C., Laurencin C.T. (2014). Small-Molecule Based Musculoskeletal Regenerative Engineering. Trends Biotechnol..

[180] Vrijens K., Lin W., Cui J., Farmer D., Low J., Pronier E., Zeng F.Y., Shelat A.A., Guy K., Taylor M.R. (2013). Identification of Small Molecule Activators of BMP Signaling. PLoS ONE.

[181] Kowalczewski C.J., Saul J.M. (2018). Biomaterials for the Delivery of Growth Factors and Other Therapeutic Agents in Tissue Engineering Approaches to Bone Regeneration. Front. Pharmacol..

[182] Lozano D., Manzano M., Doadrio J.C., Salinas A.J., Vallet-Regí M., Gómez-Barrena E., Esbrit P. (2010). Osteostatin-Loaded Bioceramics Stimulate Osteoblastic Growth and Differentiation. Acta Biomater..

[183] Pérez R., Sanchez-Salcedo S., Lozano D., Heras C., Esbrit P., Vallet-Regí M., Salinas A.J. (2018). Osteogenic Effect of ZnO-Mesoporous Glasses Loaded with Osteostatin. Nanomaterials.

[184] Teng F., Yu D., Wei L., Su N., Liu Y. (2019). Preclinical Application of Recombinant Human Bone Morphogenetic Protein 2 on Bone Substitutes for Vertical Bone Augmentation: A Systematic Review and Meta-Analysis. J. Prosthet. Dent..

[185] Kuroda Y., Kawai T., Goto K., Matsuda S. (2019). Clinical Application of Injectable Growth Factor for Bone Regeneration: A Systematic Review. Inflamm. Regen..

[186] Berkmann J.C., Herrera Martin A.X., Pontremoli C., Zheng K., Bucher C.H., Ellinghaus A., Boccaccini A.R., Fiorilli S., Brovarone C.V., Duda G.N. (2020). In Vivo Validation of Spray-Dried Mesoporous Bioactive Glass Microspheres Acting as Prolonged Local Release Systems for Bmp-2 to Support Bone Regeneration. Pharmaceutics.

[187] Kim T.H., Singh R.K., Kang M.S., Kim J.H., Kim H.W. (2016). Gene Delivery Nanocarriers of Bioactive Glass with Unique Potential to Load BMP2 Plasmid DNA and to Internalize into Mesenchymal Stem Cells for Osteogenesis and Bone Regeneration. Nanoscale.

[188] Dai C., Guo H., Lu J., Shi J., Wei J., Liu C. (2011). Osteogenic Evaluation of Calcium/Magnesium-Doped Mesoporous Silica Scaffold with Incorporation of RhBMP-2 by Synchrotron Radiation-Based ΜCT. Biomaterials.

[189] Xiao J., Luo H., Ao H., Huang Y., Yao F., Zhang Q., Wan Y. (2019). A RhBMP-2-Loaded Three-Dimensional Mesoporous Bioactive Glass Nanotubular Scaffold Prepared from Bacterial Cellulose. Colloids Surf. A Physicochem. Eng. Asp..

[190] Xin T., Mao J., Liu L., Tang J., Wu L., Yu X., Gu Y., Cui W., Chen L. (2020). Programmed Sustained Release of Recombinant Human Bone Morphogenetic Protein-2 and Inorganic Ion Composite Hydrogel as Artificial Periosteum. ACS Appl. Mater. Interfaces.

[191] Schumacher M., Reither L., Thomas J., Kampschulte M., Gbureck U., Lode A., Gelinsky M. (2017). Calcium Phosphate Bone Cement/Mesoporous Bioactive Glass Composites for Controlled Growth Factor Delivery. Biomater. Sci..

[192] Wu C., Fan W., Chang J., Xiao Y. (2013). Mesoporous Bioactive Glass Scaffolds for Efficient Delivery of Vascular Endothelial Growth Factor. J. Biomater. Appl..

[193] Zhou Y., Shi M., Jones J.R., Chen Z., Chang J., Wu C., Xiao Y. (2017). Strategies to Direct Vascularisation Using Mesoporous Bioactive Glass-Based Biomaterials for Bone Regeneration. Int. Mater. Rev..

[194] Schumacher M., Habibović P., Van Rijt S. (2022). Peptide-Modified Nano-Bioactive Glass for Targeted Immobilization of Native VEGF. ACS Appl. Mater. Interfaces.

[195] Kaya S., Cresswell M., Boccaccini A.R. (2018). Mesoporous Silica-Based Bioactive Glasses for Antibiotic-Free Antibacterial Applications. Mater. Sci. Eng. C.

[196] Kargozar S., Montazerian M., Hamzehlou S., Kim H.W., Baino F. (2018). Mesoporous Bioactive Glasses: Promising Platforms for Antibacterial Strategies. Acta Biomater..

[197] Tabia Z., El Mabrouk K., Bricha M., Nouneh K. (2019). Mesoporous Bioactive Glass Nanoparticles Doped with Magnesium: Drug Delivery and Acellular: In Vitro Bioactivity. RSC Adv..

[198] Polo L., Gómez-Cerezo N., García-Fernández A., Aznar E., Vivancos J.L., Arcos D., Vallet-Regí M., Martínez-Máñez R. (2018). Mesoporous Bioactive Glasses Equipped with Stimuli-Responsive Molecular Gates for Controlled Delivery of Levofloxacin against Bacteria. Chem.-Eur. J..

[199] Pouroutzidou G.K., Liverani L., Theocharidou A., Tsamesidis I., Lazaridou M., Christodoulou E., Beketova A., Pappa C., Triantafyllidis K.S., Anastasiou A.D. (2021). Article Synthesis and Characterization of Mesoporous Mg-and Sr-Doped Nanoparticles for Moxifloxacin Drug Delivery in Promising Tissue Engineering Applications. Int. J. Mol. Sci..

[200] El-Kady A.M., Farag M.M., El-Rashedi A.M.I. (2016). Bioactive Glass Nanoparticles Designed for Multiple Deliveries of Lithium Ions and Drugs: Curative and Restorative Bone Treatment. Eur. J. Pharm. Sci..

[201] Seedher N., Agarwal P. (2010). Effect of Metal Ions on Some Pharmacologically Relevant Interactions Involving Fl Uoroquinolone Antibiotics. Drug Metabol. Drug Interact..

[202] Epsley S., Tadros S., Farid A., Kargilis D., Mehta S., Rajapakse C.S. (2021). The Effect of Inflammation on Bone. Front. Physiol..

[203] Majumdar S., Hira S.K., Tripathi H., Kumar A.S., Manna P.P., Singh S.P., Krishnamurthy S. (2021). Synthesis and Characterization of Barium-Doped Bioactive Glass with Potential Anti-Inflammatory Activity. Ceram. Int..

[204] Chitra S., Bargavi P., Balasubramaniam M., Chandran R.R., Balakumar S. (2020). Impact of Copper on In-Vitro Biomineralization, Drug Release Efficacy and Antimicrobial Properties of Bioactive Glasses. Mater. Sci. Eng. C.

[205] Zhang X., Zhang J., Shi B. (2014). Mesoporous Bioglass/Silk Fibroin Scaffolds as a Drug Delivery System: Fabrication, Drug Loading and Release in Vitro and Repair Calvarial Defects in Vivo. J. Wuhan Univ. Technol. Mater. Sci. Ed..

[206] Mo Y., Zhao F., Lin Z., Cao X., Chen D., Chen X. (2022). Local Delivery of Naringin in Beta-Cyclodextrin Modified Mesoporous Bioactive Glass Promotes Bone Regeneration: From Anti-Inflammatory to Synergistic Osteogenesis and Osteoclastogenesis. Biomater. Sci..

[207] Wang X., Wang G., Zhang Y. (2017). Research on the Biological Activity and Doxorubicin Release Behavior in Vitro of Mesoporous Bioactive SiO 2 -CaO-P 2 O 5 Glass Nanospheres. Appl. Surf. Sci..

[208] Wang X., Zhang Y., Lin C., Zhong W. (2017). Sol-Gel Derived Terbium-Containing Mesoporous Bioactive Glasses Nanospheres: In Vitro Hydroxyapatite Formation and Drug Delivery. Colloids Surf. B Biointerfaces.

[209] Zhang Y., Wang X., Su Y., Chen D., Zhong W. (2016). A Doxorubicin Delivery System: Samarium/Mesoporous Bioactive Glass/Alginate Composite Microspheres. Mater. Sci. Eng. C.

[210] Wang X., Zhang Y., Ma Y., Chen D., Yang H., Li M. (2016). Selenium-Containing Mesoporous Bioactive Glass Particles: Physicochemical and Drug Delivery Properties. Ceram. Int..

[211] Bains R., Sharma P., Mir R.A., Jeet S., Kaur G., Pandey O.P. (2018). Influence of CuO/MgO Ratio on the Gene Expression, Cytocompatibilty, and Antibacterial/Anticancerous/Analgesic Drug Loading Kinetics for (15-x) CuO-XMgO-10P_2_O_5_-60SiO_2_-10CaO-5ZnO (2.5 ≤ x ≤ 12.5) Mesoporous Bioactive Glasses. J. Biomed. Mater. Res. Part A.

[212] Shoaib M., Ur Rahman M.S., Saeed A., Naseer M.M. (2018). Mesoporous Bioactive Glass-Polyurethane Nanocomposites as Reservoirs for Sustained Drug Delivery. Colloids Surf. B Biointerfaces.

[213] Shruti S., Salinas A.J., Ferrari E., Malavasi G., Lusvardi G., Doadrio A.L., Menabue L., Vallet-Regí M. (2013). Curcumin Release from Cerium, Gallium and Zinc Containing Mesoporous Bioactive Glasses. Microporous Mesoporous Mater..

[214] Garg S., Thakur S., Gupta A., Kaur G., Pandey O.P. (2017). Antibacterial and Anticancerous Drug Loading Kinetics for (10-x)CuO-XZnO-20CaO-60SiO_2_-10P_2_O_5_ (2 ≤ x ≤ 8) Mesoporous Bioactive Glasses. J. Mater. Sci. Mater. Med..

[215] Ur Rahman M.S., Tahir M.A., Noreen S., Yasir M., Khan M.B., Mahmood T., Bahadur A., Shoaib M. (2020). Osteogenic Silver Oxide Doped Mesoporous Bioactive Glass for Controlled Release of Doxorubicin against Bone Cancer Cell Line (MG-63): In Vitro and in Vivo Cytotoxicity Evaluation. Ceram. Int..

[216] Hu M., Fang J., Zhang Y., Wang X., Zhong W., Zhou Z. (2020). Design and Evaluation a Kind of Functional Biomaterial for Bone Tissue Engineering: Selenium/Mesoporous Bioactive Glass Nanospheres. J. Colloid Interface Sci..

[217] Zhang Y., Hu M., Wang X., Zhou Z., Liu Y. (2018). Design and Evaluation of Europium Containing Mesoporous Bioactive Glass Nanospheres: Doxorubicin Release Kinetics and Inhibitory Effect on Osteosarcoma MG 63 Cells. Nanomaterials.

[218] Sui B., Liu X., Sun J. (2018). Dual-Functional Dendritic Mesoporous Bioactive Glass Nanospheres for Calcium Influx-Mediated Specific Tumor Suppression and Controlled Drug Delivery in Vivo. ACS Appl. Mater. Interfaces.

[219] Das M.P., Pandey G., Neppolian B., Das J. (2021). Design of Poly-L-Glutamic Acid Embedded Mesoporous Bioactive Glass Nanospheres for PH-Stimulated Chemotherapeutic Drug Delivery and Antibacterial Susceptibility. Colloids Surf. B Biointerfaces.

[220] Singh R.K., Kurian A.G., Patel K.D., Mandakhbayar N., Lee N.H., Knowles J.C., Lee J.H., Kim H.W. (2020). Label-Free Fluorescent Mesoporous Bioglass for Drug Delivery, Optical Triple-Mode Imaging, and Photothermal/Photodynamic Synergistic Cancer Therapy. ACS Appl. Bio Mater..

[221] Chen S.Y., Chou P.F., Chan W.K., Lin H.M. (2017). Preparation and Characterization of Mesoporous Bioactive Glass from Agricultural Waste Rice Husk for Targeted Anticancer Drug Delivery. Ceram. Int..

[222] Shoaib M., Saeed A., Rahman M.S.U., Naseer M.M. (2017). Mesoporous Nano-Bioglass Designed for the Release of Imatinib and in Vitro Inhibitory Effects on Cancer Cells. Mater. Sci. Eng. C.

[223] Nawaz Q., Fuentes-Chandía M., Tharmalingam V., Ur Rehman M.A., Leal-Egaña A., Boccaccini A.R. (2020). Silibinin Releasing Mesoporous Bioactive Glass Nanoparticles with Potential for Breast Cancer Therapy. Ceram. Int..

[224] Rahman M.S.U., Tahir M.A., Noreen S., Yasir M., Ahmad I., Khan M.B., Ali K.W., Shoaib M., Bahadur A., Iqbal S. (2020). Magnetic Mesoporous Bioactive Glass for Synergetic Use in Bone Regeneration, Hyperthermia Treatment, and Controlled Drug Delivery. RSC Adv..

